# The Effects of Dietary Intervention on HIV Dyslipidaemia: A Systematic Review and Meta-Analysis

**DOI:** 10.1371/journal.pone.0038121

**Published:** 2012-06-11

**Authors:** Clare Stradling, Yen-Fu Chen, Tracy Russell, Martin Connock, G. Neil Thomas, Shahrad Taheri

**Affiliations:** 1 HIV Metabolic Clinic, Heart of England NHS Foundation Trust, Birmingham, United Kingdom; 2 Birmingham and Black Country NIHR CLAHRC, University of Birmingham, Birmingham, United Kingdom; 3 Dietetic Department, Western General Hospital, Edinburgh, United Kingdom; 4 Warwick Evidence, Warwick Medical School, University of Warwick, Coventry, United Kingdom; 5 School of Public Health, Epidemiology and Biostatistics, University of Birmingham, Birmingham, United Kingdom; 6 School of Clinical and Experimental Medicine, University of Birmingham, Birmingham, United Kingdom; Rush University, United States of America

## Abstract

**Background:**

Efficacy of dietary intervention for treatment and prevention of HIV-related lipid disturbances has not been well established.

**Methods:**

We conducted a systematic search of electronic databases supplemented with manual searches and conference abstracts, without language restriction. All randomised controlled trials (RCTs) with blood lipid outcomes, involving dietary intervention or supplementation for the treatment or prevention of adult HIV dyslipidaemia, versus no or other intervention were included. Two authors using predefined data fields, including study quality indicators, extracted data independently.

**Results:**

Eighteen studies (n = 873) met our inclusion criteria. Seven RCTs for omega-3 supplementation (n = 372), and four RCTs for dietary intervention (n = 201) were meta-analysed using random-effects models. Mild statistical heterogeneity was observed. Dietary intervention reduced triglyceride levels by −0·46 mmol/l (95%CI: −0·85 to −0·07 mmol/l) compared to control. Omega-3 supplementation reduced triglyceride levels by −1.12 mmol/l, (95%CI: −1·57 to −0·67 mmol/l) and total cholesterol, −0·36 mmol/l (95%CI: −0·67 to −0·05 mmol/l) compared to placebo/control.

**Conclusions:**

Both omega-3 supplementation and dietary intervention reduced triglyceride level, with the latter possibly to a smaller extent. While dietary interventions are beneficial, more stringent dietary approaches may be necessary to fully address lipid disturbances in HIV patients.

**Trial Registration:**

PROSPERO 2011:CRD42011001329.

## Introduction

While survival with HIV has increased dramatically with highly active antiretroviral therapy (ART), affected individuals are experiencing metabolic complications including insulin resistance and dyslipidaemia, that ultimately translate to increased cardiovascular disease (CVD). Current guidelines recommend dietary intervention as first line treatment for HIV dyslipidaemia, [1], [2], [3] based on evidence from the general population, where it has been shown to reduce CVD risk and mortality. [4], [5] Whether these findings can be extrapolated to the HIV population on ART is unknown. If dietary interventions were effective, CVD risk can be reduced through behaviour modification reducing toxicity and pill burden accompanying lipid-lowering medication (LLM).

Narrative reviews have examined the broader management of HIV dyslipidaemia including drug intervention, [6] and the effect of nutritional support and exercise on body composition. [7] Almeida et al examined dietary intervention from observation and intervention studies, concluding that there was little evidence for effectiveness of dietary interventions for HIV dyslipidaemia. [8] We hypothesized that dietary interventions have a beneficial effect on HIV dyslipidaemia and carried out a systematic review and meta-analysis of randomised controlled trials (RCTs) assessing the efficacy of dietary interventions or supplementation for HIV dyslipidaemia.

## Methods

Current guidelines for systematic reviews were followed [9] including protocol registration (PROSPERO 2011:CRD42011001329).

### Search Strategy

A comprehensive search was conducted using a combination of MeSH and free text terms incorporating the population (HIV infected adults), intervention (any dietary therapy or supplements) and outcome (cardiovascular or dyslipidaemia), limited to human studies and clinical trials, up to 15 March 2012 on databases from Medline (OVID from 1950), AMED (from 1985), CINAHL (from 1981), EMBASE (from 1988), and up to 31 May 2011 on OpenSIGLE (from 1980) and Cochrane Library, and clinical trial registries including the World Health Organisation and National Institutes of Health (Table S1). No language restrictions were used. The International AIDS Conference (2001 to 2010) and Conference on Retroviruses and Opportunistic Infections (1997 to 2010) websites were searched by use of the term ‘diet’.

### Selection Criteria

Articles resulting from these searches and relevant references cited in those articles were screened and assessed independently by two reviewers (CS and TR) for eligibility. All RCTs involving dietary intervention or nutritional supplementation given for prevention or treatment of HIV metabolic disturbances in adults were included.

All-cause mortality or cardiac events were preferred as primary outcomes, however frequent reporting of these was not anticipated therefore surrogate markers such as change in serum lipids, were also sought. The control group was usual diet, no intervention or placebo; however, head to head intervention studies were also included. Studies lacking a control, or dietary intervention, or those whose primary focus was not prevention or treatment of metabolic disturbances were excluded. Duplicate publications were also excluded.

### Validity Assessment

Study level risk of bias was assessed (independently in duplicate) using the Cochrane Collaboration 6-item domain based evaluation (version 5·0). [10] Diet specific factors, such as intervention adherence and potential confounding lifestyle factors (smoking, exercise, alcohol) were incorporated into the performance bias section (see Figure S1). A score of −1, 0, +1, was assigned to judgments ‘high’, ‘unclear’, and ‘low’ risk, respectively, for the purpose of study quality categorisation used to inform sensitivity analysis. Score consensus was reached following discussion of each study.

### Data Extraction

Piloted forms and duplicate standardised databases were used by two reviewers to independently extract the following data: study characteristics (design, setting, enrolment date, sample size, eligibility criteria, quality, funding, ethics), population studied (baseline characteristics – lipids, body mass index, smoking, ART, ethnicity, age, gender, LLM), therapeutic interventions (duration, intensity, advice specifics regarding diet, exercise, smoking and adherence to it, comparison of intakes), and control regimen. Outcomes assessed included serum total cholesterol (total cholesterol), high-density lipoprotein cholesterol (HDL-cholesterol), Low-density lipoprotein cholesterol (LDL-cholesterol), and triglyceride (TG) levels. Discrepancies were resolved by discussion and comparison with the primary study report.

### Data Analysis

No studies reported clinical endpoints, therefore outcome data (baseline and final mean values with standard deviation, SD) for total cholesterol, LDL-cholesterol, HDL-cholesterol, and triglyceride was extracted and converted to SI units. Authors were contacted for missing outcome data or raw data where non-parametric outcomes were presented and to clarify discrepancies. In absence of author response, the SD of the outcome mean for intervention and control groups was estimated from the respective standard error or confidence intervals if reported, or the p value of the mean difference between groups. In absence of reporting of final means, or where there was a significant difference between intervention and control group baseline values, change from baseline was used in the meta-analysis. Where clinical homogeneity allowed, effect sizes were pooled, and weighted by inverse variance of each study’s effect estimate. Studies reporting insufficient data were excluded from meta-analysis.

The principal summary measure was difference in mean lipid levels (total cholesterol, LDL-cholesterol, HDL-cholesterol, triglyceride) post-intervention between groups. Due to broad eligibility criteria regarding dietary intervention, clinical heterogeneity was expected and a random effects model planned. Statistical heterogeneity was quantified using I^2^ statistic and assessed for strength of evidence using the chi-squared test. Subgroup analysis was planned *a priori* where heterogeneity was anticipated between studies, arising from variations in baseline characteristics (normal or elevated lipid levels, use of LLM, use of ART), treatment type and duration, and comparators. Dose related response for omega-3 supplements was explored using meta-regression. Sensitivity analyses were pre-planned to assess the effect on the pooled result of exclusion of studies with small sample size (defined as less than 30 participants), studies of low quality, based on the risk of bias score (<2), unpublished studies, and inclusion of change scores within the analysis based on final values. Publication bias was explored using funnel plots where numbers of studies allowed (>10). Data were analyzed using RevMan 5·0 (The Cochrane Collaboration, UK) [11] and STATA 10 (StataCorp LP, Texas, USA).

## Results

**Figure 1:**
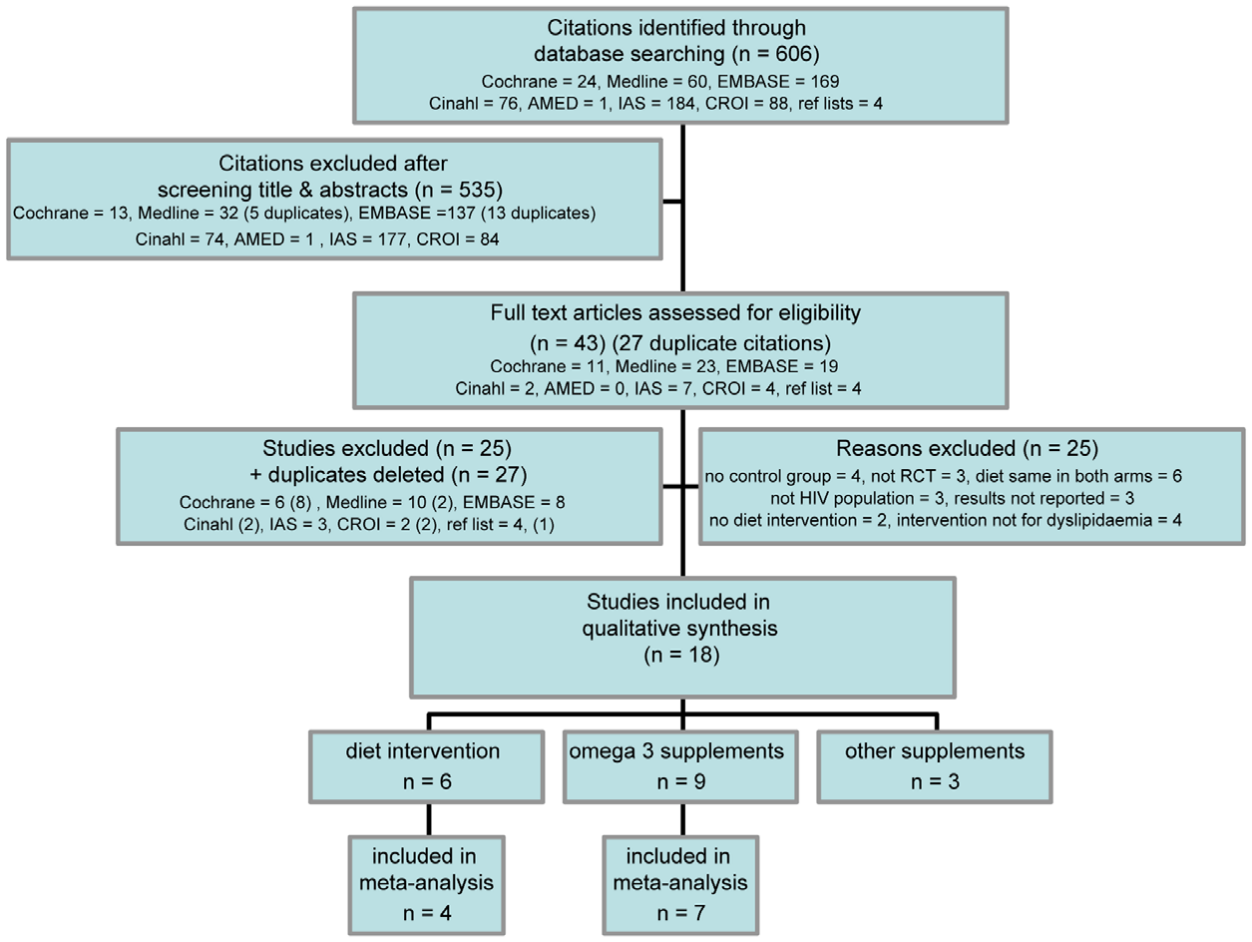
Prisma flow diagram of study selection and exclusion.

Literature searches retrieved 606 citations; after screening and exclusion, 43 articles were further scrutinised. (Excluded studies are listed in Figure S2). Eighteen primary studies met selection criteria (Figure 1). Three of these were from conference proceedings, available only in abstract form [12], [13] or with additional information from authors [14].

### Study Characteristics

Table 1 shows key characteristics of included studies. Of 18 RCTs, seven were placebo-controlled, [12], [15], [16], [17], [18], [19], [20] and two used an alternative comparator, fenofibrate [21] or Mediterranean diet (MD). [22] All studies were in English, mainly from North America (n = 10), with some from Europe, [15], [17], [20] east Asia, [13], [16], [22] and South America. [14], [23] Median study duration was 12 weeks (range: 8–16 weeks) for tablet (omega-3/other supplementation) studies and 26 weeks (range: 16–52 weeks) for diet studies. Sample sizes ranged from 11–120 participants (median: 46).

**Table 1:** Study characteristics.

STUDY	COUNTRY	n/N	INTERVENTION	n/N	CONTROL	DURATION	OUTCOME MEASURE
**Omega 3 supplementation**							
Peters 2012	Europe	23/25	2 g Omacor bd (4×460 mg EPA + 380 mgDHA + 4 mg vitamin E)	25/25	Matching placebo	12 weeks	% change in TG
Thusgaard 2009	Denmark	25/26	2 Omacor bd (4×460 mg EPA + 380 mgDHA + 4 mg vitamin E)	23/25	2 corn oil bd	12 weeks	TG
Carter 2006	Australia	5/5	3 Maxepa tds (9×EPA 180 mg + DHA 120 mg) +diet	6/6	Identical placebo + diet	6 wks diet +8 wkstablets	TG +total cholesterol
DeTruchis 2007	France	58/60	Maxepa tds (EPA 1080 mg + DHA 720 mg) + diet	62/62	Identical placebo + diet	4 wk diet +8 wktablets	% change in TG
Woods 2009	United States	21/28	5 Omega Rx (5×EPA 400 mg + DHA 200 mg) + 100 mg/d Vitamin E + diet (including 3 g/d omega-3)	26/26	usual diet; no advice	13 weeks	TG at week 3 & 13
Wohl 2005	United States	Wk 4 24/26 (wk 16 22/26)	Fish oil (1750 mg EPA + 1150 mg DHA + 10 mgVitamin E) + diet + exercise	Wk 4 20/26 (Wk 16 19/26)	Diet + exercise	16 wks	TG at wk 4
Baril 2007	Canada	26/30	1 g salmon oil tds (3×180 mg EPA + 120 mg DHA)	31/36	No placebo	24 weeks (but Ax at12 wks)	Change in TG
Peabody 2002§	Canada	∼14¶	300 mg omega 3 daily	∼14¶	Olive oil placebo	4 wk diet +8 wk tablets	TG, LDL, HDL, total cholesterol
Gerber 2008	United States	47/50	3 g fish oil bd (6×500 mg EPA + 310 mgDHA + 13 mg Vitamin E)	48/50	160 mg fenofibrate od(combination Rx if TG>200 mg/dl)	8 wks +8 wks	Response at wk 18 TG<200 mg/dl on combination Rx
**Dietary intervention**							
Fitch 2006	United States	12/16	NCEP style diet with weekly one-to-one counseling sessions + 3 hr exercise/wk = 10,000 steps/d	16/18	usual diet; one session withdietitian at baseline	6 months	change in waist circumference at week 24
Sanchez 2006 §	Argentina	12/16	NCEP style diet with sessions every 3 months + supervised resistance and aerobic exercise 3 times a week	10/15	usual diet + unsupervisedresistance and aerobic exercise3 times a week	6 months	within and between group change in total cholesterol, HDL-, glucose, insulin, limb fat assessed by DEXA at week 24
Lazzaretti 2012	Brazil	43/45	Phase II NCEP diet with sessions every 3 months	40/45	usual diet; one session with dietitian at baseline	12 months	change in lipids at week 52
Balasubramanyam 2011	United States	38/43	NCEP style diet with sessions every 2 months + daily internet support + supervised exercise 3×week	30/41	Usual diet; booklet on healthyheart diet	6 months	Change in TG, HDL, non-HDL cholesterol
Ng 2011	Hong Kong	25/25	Low fat diet (NCEP style) with sessions every 3 months	23/23	Mediterranean diet (low sat fat+3 items from list) with sessions every3 months	12 months	total cholesterol TG, WC, BMI, triceps skinfold thickness
Thanasilp 2010§	Thailand	19	SMCCP Symptom management model + diet +yoga + Qi-gong	23	Routine nursing care	4 months	LDL-, HDL-cholesterol
**Other tablet supplementation**							
Hadigan 2006	United States	11/11	250 mg acipimox tds	11/12	Identical placebo	3 months	TG
Chow 2010	United States	10	1500 mg extended release niacin	9	No placebo	12 weeks	Flow mediated dilatation of branchial artery, HDL-
Aghdassi 2010	Canada	23/26	400 ug chromium (2 pills bd)	23/26	Identical placebo	16 wks	Insulin resistance (HOMA-IR)

Characteristics of the eighteen studies included in the qualitative synthesis: setting, sample size, intervention, control, and duration.

* median value.

§ unpublished data.

¶ estimated, not reported by study.

n  =  number of participants analysed.

N  =  number randomised.

bd  =  twice daily.

tds  =  three times a day.

EPA  =  eicosapentaenoic acid.

DHA  =  docosahexaenoic acid.

NCEP  =  National Cholesterol Education Programme [26].

wk  =  week.

Ax  =  assessment.

DEXA  =  dual-energy x-ray absorptiometry.

The majority of studies (12/18) involved specific nutrient supplementation: nine with omega-3 (daily dose range: 900–4860 mg total eicosapentaenoic acid [EPA] and docosahexaenoic acid [DHA]; Table S2), three of which also included vitamin E. [20], [21], [24] Others employed a form of nicotinic acid [18], [25] or chromium. [19].

Six studies evaluated dietary interventions. Due to lack of full text publication, very little was reported on dietary components of the symptom management model in one of the studies, [13] but other studies were based on advice following the NCEP ATPIII dietary recommendations [26]. The specifics of these five diet studies varied, such as level of fibre, and inclusion of exercise, [14], [23], [27], [28] whilst two studies also provided meals for 2–3 weeks [24], [28] (Table S3). Intervention intensity varied from daily online support [28] or weekly individual counselling for six months, [27] to fortnightly [14] and three-monthly sessions. [23] Control groups in diet studies received usual care.

Most of the 873 participants in included studies were men (Table 2). Ethnicity ranged from 100% Chinese [22] and 100% Thai, [13] to most studies containing both African and Caucasian participants. Mean age ranged from 38–50 years. Mean baseline fasting triglyceride levels ranged 1·51–7·54 mmol/l and total cholesterol 3·94–6·24 mmol/l. Participants were generally on stable ART with hypertriglyceridaemia, except the Brazilian study where patients naïve to ART with and without dietary advice were observed for dyslipidaemia development. [23] The Chinese study mainly recruited newly diagnosed HIV patients. [22] Use of ART regimens containing protease inhibitors (PI) ranged from 14–82%. Studies mainly assessed fasting blood lipids as primary outcome; in two studies, blood lipids were secondary outcomes after waist circumference [27] and insulin resistance. [19].

**Table 2:** Participant characteristics.

STUDY	POPULATION		ELIGIBILITY CRITERIA	DRUGS		BASELINE LIPIDS (mmol/l)	
	Gender, mean age, ethnicity	Mean BMI, Smokers		ART	LLM	TG	total cholesterol
**Omega 3 supplementation**							
Peters 2012	98% men 45 yrs 90% white	BMI 24	Fasting TG between 3.4–11.3 mmol/l on low cholesterol diet, avoiding excess alcohol	Stable ART >3 months 52% PIs 46% RTV	All on fibrate or niacin	4.9	6.3
Thusgaard 2009	78% men 45 yrs 86% white	BMI 24.7 31% smokers	All patients on ART	>3 months ART 47% PIs	8%	1.67	5.45
Carter 2006	100% men48 yrs	BMI 24	total cholesterol <6.5 mmol/l; TG 3.5–10 mmol/l	Stable ART >6 months82% PIs	18%	5.06	5.77
DeTruchis 2007	89% men 46 yrs	BMI 23	TG ≥3.43 mmol/l + TG 2–10 g/l after 4 week TG lowering diet; glucose ≤6.6 mmol/l, alcohol ≤20 g/d	Stable ART ≥2 months72% PIs	Nil	4.5	NR
Woods 2009	80% men 47 yrs* 50% white	BMI 25*	BMI 19–30+ TG >1.69 mmol/l or insulin resistance	87% on ART 63%PIs 50% RTV	Nil	2.01*	5.08*
Wohl 2005	90% men 44 yrs 46% white	BMI 27	TG >2.26 mmol/l	Stable ART >3 months43% RTV	Yes %NR	5.44	6.23
Baril 2007	98% men, 49 yrs, 95% white	72% smokers (current or past) 14% DM	triglyceride 6–11 mmol/l, or triglyceride 2–6 mmol/l with total cholesterol:HDL-cholesterol ≥6	Stable ART >6 months 60% PIs	59%	5	6.05
Peabody 2002§	75% men 42 yrs	BMI 25	triglyceride >3 mmol/l	On ART	NR	4.42	5.71
Gerber 2008	93% men 43 yrs* 57% white		triglyceride ≥4.5 mmol/l + LDL-cholesterol ≤4.1 mmol/lAdherence to lipid lowering diet and exercise for 28 d pre screening	ART >3 months 39% PIs	Nil	7.54*	6.33*
**Dietary intervention**							
Fitch 2006	% NR 45 yrs,32% white,	BMI 32, 45% smokers	Metabolic syndrome = 3/5 of: raised triglyceride, WC, or BP, fasting glucose, low HDL-cholesterol	stable ART >1 month 53% PIs	9%	2.48	5.14
Sanchez 2006§	74% men 42 yrs	BMI 25 52% smokers	Lipodystrophy + no alcohol abuse + no Family History of dyslipidaemia	Stable ART >6 months	Nil	2.62§	5.57§
Lazzaretti 2012	37% men 38 yrs	BMI 24 27% smokers	No ART, no LLM, no history of CVD/dyslipidaemia (mean CD4 177)	naïve to ART 19%started PIs	3% started	1.52	3.94§
Balasubramanyam 2011	91% men 45 yrs 38% white	BMI 27 62% Hx of smoking	Triglycerides between 1.7–11.3 mmol/l + BMI 19–35+ no LLM + CD4>100	Stable ART >6 months 72% PIs	Nil	3.62	5.63
Ng 2011	77% men 41 yrs 0% white	BMI 22	Not previously received diet advice + stable with HIV diagnosis and no current illness(many newly diagnosed and not stable)	79% on ART 17% PIs	Yes %NR	1.99	4.69
Thanasilp 2010§	100% women		On ART	100% on ART			
**Other tablet supplementation**							
Hadigan 2006	74% men 46 yrs	BMI 27	Lipodystrophy + triglyceride >1.69 mmol/l + no alcohol abuse	Stable ART >3 months 52% PIs	Nil	2.99	5.23
Chow 2010	89% men 51 yrs* 53% white	BMI 25* 21% smokers 37% ex smokers	HDL-cholesterol <1.04 mmol/l + LDL-cholesterol <3.37 mmol/l	Stable ART ≥6 months 42% PIs	Nil	1.76	4.67
Aghdassi 2010	96% men 48 yrs	BMI 26 33%smokers	One metabolic abnormality (raised glucose, triglyceride, total cholesterol or low HDL-cholesterol, self reported LD) + HOMA-IR >2.5 (fasting glucose x insulin)	Stable ART >3 months 65% PIs	31%	2.64	4.97

Characteristics of the eighteen studies included in the qualitative synthesis: Population, eligibility criteria, baseline lipid levels, use of antiretroviral and lipid lowering drugs.

NR  =  not recorded.

LLM  =  lipid modification medication.

ART  =  antiretroviral therapy.

PIs  =  protease inhibitors.

RTV  =  ritonavir.

BMI  =  body mass index.

DM  =  diabetic.

CVD  =  cardiovascular disease.

HOMA-IR  =  insulin resistance.

Conversion factor used: x 0.01129 for mg/dl to mmol/l.

### Risk of Bias

**Figure 2:**
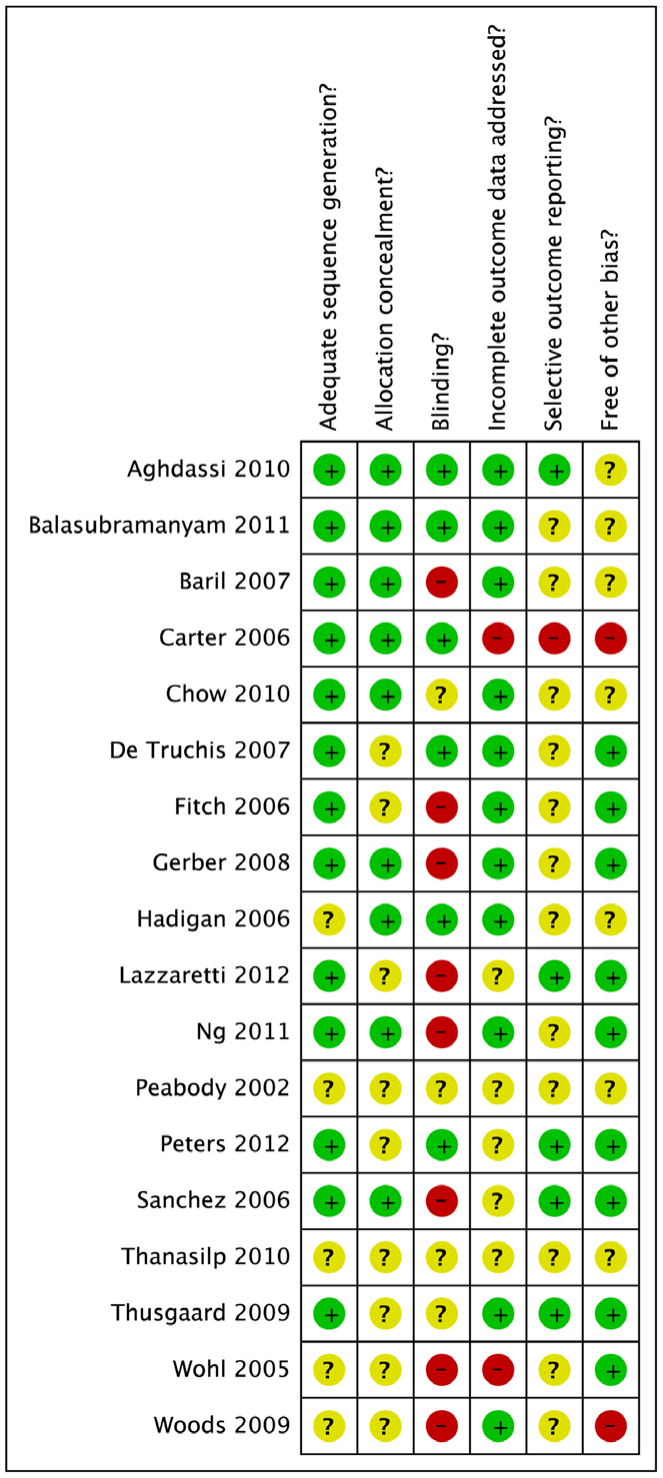
Risk of bias summary. Review authors’ judgements about each risk of bias item for each included study.

Most studies were classified as low to moderate risk for selection, attrition, reporting and performance bias (Figure 2). Lack of blinding was the most common reason for high bias risk (Figure S3) and in diet studies this might have resulted in the control group also changing their diet due to trial participation, potentially producing bias against observing an effect of the intervention. Other main reasons for high bias risk were lack of information about attrition, lack of intention to treat analysis or power calculation, and potential imbalance at baseline in use of alcohol, cigarettes, or exercise. Randomisation information lacked clarity in half of studies.

### Diet Studies

**Figure 3:**
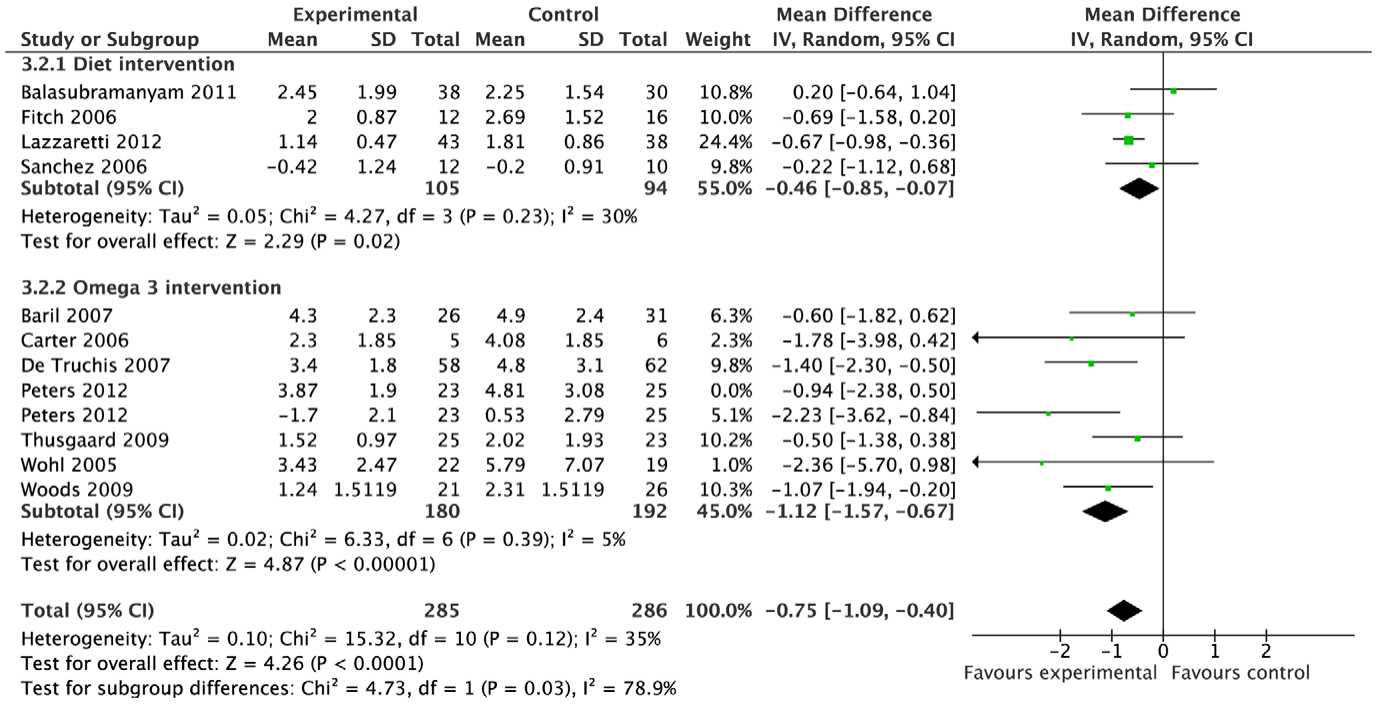
Forest plot for effect of various dietary interventions on triglyceride levels (mmol/l). Studies are ranked from low to high baseline triglyceride levels. Illustrates weighted mean difference in triglyceride levels between dietary intervention or omega-3 supplementation and control group.

Among the six diet studies, two studies were excluded from meta-analysis due to lack of data, [13] and head-to-head design. [22] The pooled estimate for four studies (n = 201) [14], [23], [27], [28] showed no significant differences between dietary intervention and control groups for total cholesterol (0·01 mmol/l; 95%CI −0·71 to 0·73, p = 0·97, I^2^ = 85%), HDL-cholesterol (0·11 mmol/l; 95%CI −0·01 to 0·22, p = 0·07, I^2^ = 33%), and LDL-cholesterol (3 studies; −0·01 mmol/l, 95%CI −0·81 to 0·79 mmol/l, p = 0·98, I^2^ = 87%); and there was evidence of substantial statistical heterogeneity. Reduction in fasting triglycerides was significantly greater with dietary intervention versus controls, the random effects point estimate was: WMD −0·46 mmol/l, 95%CI −0·85 to −0·07 mmol/l (p = 0.02) (Figure 3) with moderate statistical heterogeneity (I^2^ = 30%).

Similar treatment effect estimates were found on sensitivity analysis with exclusion of small trials (<30 participants), [27] duration of less than six months, intervention including exercise, [14], [27], [28] change scores rather than final value, [14] unpublished trials [14], [23] and those with high bias risk [23] (data not shown).

### Omega-3

**Figure 4:**
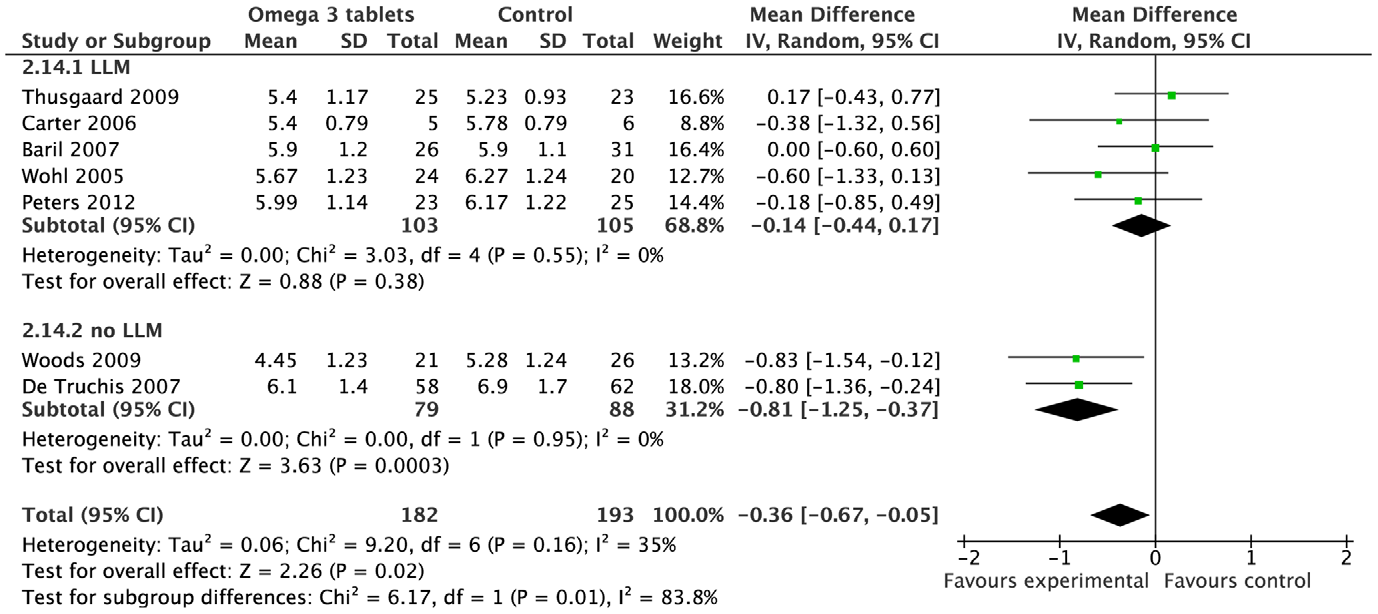
Forest plot for effect of omega-3 supplementation on cholesterol levels (mmol/l). Studies are ranked from low to high baseline cholesterol levels. Illustrates subgroup analysis of concurrent use of lipid lowering medication (LLM) on treatment effects of omega-3 supplementation on Cholesterol levels.

Of the nine omega-3 studies, two were excluded from meta-analyses due to lack of data [12] and head-to-head design [21] (Table S4). The excluded omega-3 trials illustrated extremes in both omega-3 doses and outcomes, from 13% reduction in triglyceride with 300 mg omega-3, [12] to 46% triglyceride reduction with 4860 mg omega-3 (versus 58% in fenofibrate comparison group). [21] Seven studies (n = 372) reported results for fasting triglycerides and total cholesterol and were included in meta-analysis. There was a significantly greater reduction in fasting triglycerides among those receiving omega-3, compared to controls; random effects point estimate −1.12 mmol/l, 95%CI −1.57 to −0.67 mmol/l (p<0·0001, I^2^ = 5%) (Figure 3). Total cholesterol reduction (−0·36 mmol/l, 95%CI −0·67 to −0·05 mmol/l, I^2^ = 35%) (Figure 4) was significant (p = 0.02) when SD imputation used the average from other studies (SD = 1.23 and 1.24) as planned a priori, but was of borderline statistical significance (p = 0·05) when alternative SD imputation (at ‘reasonably high’ value SD = 1.5) was used in sensitivity analysis.

The pooled estimate from five studies (n = 240) indicated no significant difference between intervention and control groups for LDL-cholesterol (0·10 mmol/l, 95%CI −0·15 to 0·35 mmol/l, p = 0·43, I^2^ = 0%), and the pooled estimate for 6 studies (n = 365) similarly indicated no difference for HDL-cholesterol (0·05 mmol/l, 95%CI 0·00 to 0·11 mmol/l, p = 0·07, I^2^ = 0%, Figure S5).

Sensitivity analyses showed that methodological heterogeneity did not appear to influence effect estimates; the six studies with sample sizes >30 [15], [17], [20], [24], [29], [30] and the four trials meeting quality criteria [15], [17], [20], [29] produced results similar to the overall meta-analysis (triglycerides MD −1.10 mmol/l, 95%CI −1·60 to −0·61 and −1.10 mmol/l, 95%CI −1·82 to −0·39, respectively).

Subgroup analysis, according to whether placebo was used in the trial, did not demonstrate any differences in treatment effects (test for subgroup difference, triglyceride p = 0·62, total cholesterol p = 0·89). A significant difference in treatment effect between LLM subgroups, for cholesterol only, was found (test for subgroup difference p = 0·01), with trials allowing LLM use showing no significant treatment effect (−0·14 mmol/l, 95%CI −0·44 to 0·17) compared with those excluding LLM use (−0·81 mmol/l, 95%CI −1·25 to −0·37) (Figure 4). Thus, the moderate statistical heterogeneity among omega-3 studies for reduction in total cholesterol (I^2^ = 35% as shown earlier) is likely due to the variability in exclusion criteria in relation to LLM use.

Risk stratification of studies suggested possible trends of increasing treatment effect with greater underlying risk, as defined by baseline triglyceride levels (Figure 3). Further exploration with meta-regression, however, found no evidence of a significant association between either baseline triglyceride levels (p = 0·1 and p = 0·6), omega-3 dose (p = 0·9 and p = 0·7) or proportion of participants on PI-based regimens (p = 0·4 and p = 0·6) and mean difference in triglycerides or total cholesterol, respectively, between intervention and control groups. Although the meta-regression was planned *a priori*, the small number of trials limited the statistical power of analyses, which remain exploratory.

### Publication Bias

Small statistical heterogeneity was observed in the diet and omega-3 meta-analyses for triglycerides (I^2^ = 30%, p = 0·23 and I^2^ = 5%, p = 0·39, respectively). There was some evidence of asymmetry in the funnel plot (Figure S6). Potential causes include publication bias, genuine small study effects, and statistical heterogeneity; the latter was small and an unlikely explanation. [31] Formal testing of statistical significance of the asymmetry was not undertaken because of the small number of studies.

### Other Supplementation

Three studies were excluded from the meta analyses above due to their use of other forms of supplementation, rather than omega-3 or dietary intervention. Two small studies investigated the use of nicotinic acid analogues, reporting a significant reduction in triglyceride −0.54 mmol/l versus placebo +0.65 mmol/l (p = 0.01) in one, [18] and a significant increase in HDL-cholesterol +0.08 mmol/l compared to control group −0.03 mmol/l (p = 0.04) in the other. [25] Chromium supplementation was associated with improvement in insulin resistance and a significant reduction in triglycerides −0.54 mmol/l (p = 0.03) in one study, where the effect was greater in participants with lipodystrophy −0.78±0.30 mmol/l compared to those without 0.14±0.19 mmol/l (p = 0.017) [19] (Table S4).

### Head-to-head Studies

A head-to-head study found no clinically significant difference in treatment effect between MD versus low fat diets (LFD). [22] The interventions appeared to differ in delivery rather than content. Trends for reducing total cholesterol levels with LFD, but maintaining triglyceride levels with MD were seen. Lack of study exclusion criteria resulted in a varied sample including unstable patients, those newly diagnosed with HIV or recently commenced on ART (Tables 2 and S4). [22].

## Discussion

### Findings

Dietary change is recommended to address deleterious lipid profiles predisposing to CVD in HIV patients. We did not find any studies reporting cardiovascular outcomes with dietary intervention in this patient population, therefore the objective outcome measures of serum lipids were used. Our meta-analysis revealed that a median dose of 2.8 g/d omega-3 was effective in reducing fasting triglycerides by −1.12 mmol/l over an average treatment period of 12 weeks. Likewise NCEP Therapeutic Lifestyle Changes diets (TLC) [26] reduced fasting triglyceride reduction by −0·46 mmol/l over an average treatment period of eight months in ART treated patients. Importantly, no significant effect on HDL-cholesterol or LDL-cholesterol was seen with either diet or omega-3 intervention, and only a minor reduction in total cholesterol was observed with omega-3 supplementation. These findings have good external validity coming from 5 different continents.

### Interpretation of Findings

#### Dietary Intervention

In the general population, current evidence from meta-analyses suggest that dietary intervention reduces triglycerides by −0·12 mmol/l (18·2%) with TLC style diets and exercise. [32] Previous reports of reductions of −0·17 mmol (8%) and −0·19 mmol/l (8%) with the Step I and Step II NCEP diets, respectively, may have been over estimated by outdated statistical approaches. [33] These are somewhat less than the reduction observed in our pooled estimate, presumably because of differences in baseline triglyceride levels (mean 2.20 mmol/l in the 3 HIV population diet studies versus 0.98 mmol/l in the general population studies [32]).

The lack of a beneficial effect of diet on HDL-cholesterol levels in our analysis, also observed by other reviews, [32], [33] was potentially due to the diets’ emphasis on lowering saturated fat which also reduces HDL-cholesterol. [33], [34] The positive effects of exercise on HDL-cholesterol [35], [36] may also be insufficient to override the lowering effect of diet. [32].

We observed no differences for LDL-cholesterol. This was unexpected, as previous meta-analyses in the general population have shown significant reductions in LDL-cholesterol with diet. Reasons for this are uncertain but may include lack of diet intensity and adherence. Evidence from meta-analyses suggest an incremental effect on lipid lowering with diet intensity, from an LDL-cholesterol reduction of −0·18 mmol/L (95%CI 0·1 to 0·27) with general dietary advice [37] to −0·49 mmol/l (12%) with Step I, and −0·65 mmol/l (16%) with Step II NCEP diets. [33] Recent RCTs suggest that this treatment effect may be magnified with addition of a ‘portfolio’ of cholesterol-lowering foods: plant sterols, soy protein, viscous fibre, and almonds, to produce an LDL-cholesterol reduction −1·36 mmol/l (28·6%) in feeding trials. [38] However, when performed under real-life conditions, the LDL-cholesterol reduction was −0·67 mmol/l (13·8%) due dietary adherence of only 46%. [39] Higher intensity dietary interventions may also be required in the HIV population for LDL-cholesterol reduction. Changes in LDL-cholesterol may have been limited in the included studies by adherence, as measures revealed that although significant improvements to dietary intake were made with regard to fat and fibre, levels attained did not reach the goals set [23], [24], [27](Figure S4). The significant reduction in total cholesterol observed in one of the included studies at week 3 (following controlled feeding), but not at week 13 [24] further supports the importance of dietary adherence, suggesting that dietary interventions may be difficult to implement in real-life, also seen in uncontrolled studies. [40].

Another difficulty with dietary interventions is establishing which components alter the outcome of interest and whether they act independently, synergistically or accumulatively. The magnitude of effect observed in the Woods study [24] could be explained by the dietary intervention enhancing triglyceride reduction as well as improving HDL-cholesterol levels synergistically with omega-3. It is unlikely that the addition of exercise [14], [27] would affect the outcome of dietary intervention alone, as this intensity and duration of exercise has previously proved insufficient to reduce triglycerides. [41], [42], [43], [44].

#### Omega-3

Our study supports a role for omega-3 supplementation in primary treatment of hypertriglyceridaemia in HIV patients. A systematic review in patients with diabetes (mean dose 3·5 g/d fish oil) reported similar reductions in triglycerides. [45] In primary prevention, from 47 RCTs (16,511 subjects), a mean dose 3·25 g/d and duration 24 weeks, reduced triglyceride by −0·34 mmol/l (95%CI −0·41 to −0·27) from mean baseline 2·44 mmol/l. [46] The magnitude of triglyceride reduction was significantly related to baseline triglyceride and fish-oil dose, but not duration. We observed no evidence of a dose effect, probably due to the small number of studies; however in the general population, a 5–10% reduction in triglycerides has been reported with every 1 g of EPA/DHA consumed. [47] Subgroup analysis in our review revealed significant total cholesterol reduction with omega-3 in patients who were not on statin therapy, highlighting the importance of explicit exclusion criteria in trials, as concurrent LLM likely masked the effect.

Omega-3 supplementation has a triglyceride-lowering effect that may impact on cardiac outcomes. Previously meta-analyses reported reductions in mortality and cardiovascular events [48], [49], [50] in patients with existing CVD who were taking fish oil supplements. However, they may have been subject to publication bias as inclusion of more recent trials (GISSI-HF,OMEGA, JELIS) produce pooled analyses of non-significant reduced mortality risk. [51] This suggests that doses of 1 g/d omega-3 cannot reduce mortality risk further than provided by optimised drug treatment. JELIS results suggest that the dose-dependent effect of EPA may be linked to reduction in triglycerides that is distinct from LDL-cholesterol reduction with LLM. Similarly, evidence from previous reviews that fish oil supplementation was associated with significant reduction in cardiac deaths [50] suggests that the higher dose 3–4 g/d needed to reduce triglycerides, inflammation, increase vascular reactivity, and reduce platelet function [52] may be more appropriate for primary and secondary prevention; therefore trials are required with higher doses.

### Strengths and Limitations

Previous reviews have been narrative and examined the broader management of HIV dyslipidaemia. McGoldrick et al focused on drug intervention as their search criteria and did not identify any trials concerning diet or supplements. [6] Leyes et al described 4 intervention trials, 2 open label and 2 randomised, studying only the effect of statin therapy and exercise levels, but not diet. [7] The most recent narrative review concluded that there was little evidence on effectiveness of dietary interventions for prevention and control of HIV dyslipidaemia. [8] Our systematic review is the first to employ a comprehensive search strategy and meta-analysis and demonstrates a role for dietary intervention.

While results of this review are encouraging, they must be viewed with caution due to several limitations. The small number of dietary intervention studies included in the analyses, their small sample sizes and inclusion of participants with mild dyslipidaemia limited the capacity to detect changes in lipids due to Type 2 error. Whilst statements can be made regarding the effectiveness of omega-3 supplementation and hypotheses can be proposed regarding dietary intervention, the limited number of studies available for each specific intervention do not allow us to make definitive statements about the individual effectiveness of NCEP diets, niacin or chromium.

Also, regarding validity, most studies either had some methodological weakness or had certain elements of methodology inadequately reported (Figure 2, Figure S3). All diet studies lacked blinding, which is problematic in diet studies. Additionally, it is difficult to have an appropriate control intervention. [53].

Despite lack of evidence of statistical heterogeneity in the meta-analyses for triglycerides, clinical heterogeneity was anticipated between dietary intervention studies due to variations in interpretation of the extensive NCEP ATPIII diet guidelines (Table S3). Additionally, study objectives differed, where one [23] examined dietary intervention to prevent dyslipidaemia, whilst others sought to correct dyslipidaemia/lipodystrophy. [14], [23], [27] Omega-3 studies included for meta-analysis were clinically homogeneous vis-à-vis intervention, control, duration and outcome; the main variations included differing participant ethnicities, facilitating transferability of findings, and baseline triglyceride levels or omega-3 dose, which were examined through ranking (Figure 3). The underlying causes of dyslipidaemia and whether they were due to direct HIV drug effects could not be quantified due to the variety of ART used, however, treatment regimens remained unchanged for the duration of the studies.

Another issue was the general incomplete reporting of study design and outcome data, as this hampered synthesis of included studies, requiring estimation of standard deviations in four studies. Risk of bias was difficult to assess with respect to allocation concealment, and selective outcome reporting (Figure 2). Future studies need to also include data on potential confounding factors such as other lifestyle factors (e.g. use of alcohol, tobacco, physical activity), or treatment modalities (e.g. protease inhibitors).

### Clinical Implications

Current HIV guidelines recommend diet and exercise as first line treatment. Given the limitations of available studies, our findings support a role for diet with respect to triglycerides, but not other lipids. Omega-3 supplementation was effective in lowering triglycerides and total cholesterol, but had no impact on LDL-cholesterol or HDL-cholesterol. Omega-3 supplementation is recommended as second line therapy to fibrates in American guidelines. [1] The 25% triglyceride reduction from omega-3 in our pooled analysis is nearly comparable to the 30% triglyceride reduction reported with fibrates in the general population. [54] Therefore, in ART-treated HIV patients, omega-3 may to be an alternative to fibrates, which may not be well tolerated and interact with PIs. Also, the total cholesterol reduction with omega-3 may obviate the need for statins, and their attendant side effects, in patients with primarily high triglycerides. Recent reviews have both questioned the role of omega-3 in cardiovascular event or mortality reduction [55] and reported a reduction in cardiac and overall mortality with omega-3 supplementation. [56] Specific studies are needed to examine these outcomes in ART treated HIV patients. Triglyceride levels represent an important biomarker of CVD, because of their association with atherogenic remnant particles [57] and their independent association with an increased risk of MI, seen in the prospective HIV DAD cohort. [58] However, the effect of diet on a single biomarker cannot be independently considered, and overall CVD risk should be considered.

### Conclusions

Available studies have only reported on impact of dietary intervention on surrogate lipid markers in ART-treated HIV patients. Regarding lipid markers, our meta-analysis provides evidence for a comparable clinical benefit of dietary intervention or omega-3 supplementation in reducing triglycerides, but no effects on other lipids in patients without concurrent LLM.

Our findings support the view that lifestyle interventions are a reasonable first strategy in clinical practice to improve lipid profile. Current dietary approaches, however, may be insufficient to independently tackle HIV dyslipidaemia and associated CV risk. Studies with interventions of sufficient duration and intensity, including a wide range of cholesterol lowering dietary components, focusing on foods rather than nutrients are required to elucidate the full potential of dietary intervention on lipid biomarkers and CVD. These studies would benefit from both efficacy (as treated) and effectiveness (intention to treat) analyses to address proof of principle for individual dietary components and whether they reduce CVD in clinical practice.

## Supporting Information

Figure S1 Risk of bias assessment tool.
(DOC)

Figure S2 List of excluded studies and reasons.
(DOC)

Figure S3 Risk of bias graph: review authors’ judgements about risk of bias item presented as percentages across all included studies.
(EPS)

Figure S4 Adherence to diet – Graphs to show levels of nutrients advised and consumed.
(DOC)

Figure S5 Forest plot to show effect of omega-3 supplementation on High Density Lipoprotein cholesterol levels (mmol/l).
(EPS)

Figure S6 Funnel plot to investigate possible small study effects for triglyceride.
(EPS)

Table S1 Search strategy.
(DOC)

Table S2 Fish oil doses.
(DOC)

Table S3 Dietary interventions.
(DOC)

Table S4 Results of studies not included in meta analysis.
(DOC)
