# Supplementary material for: The Effects of Dietary Intervention on HIV Dyslipidaemia: A Systematic Review and Meta-Analysis
Source: PLoS One. 2012 Jun 11;7(6):e38121. doi: 10.1371/journal.pone.0038121 (PMC3372478; doi:10.1371/journal.pone.0038121)
Supplement: Figure S1 — Risk of bias assessment tool. (DOC) [file pone.0038121.s001.doc]

**Figure S1**

**Risk of bias assessment tool**

For further detail see Table 8.5a & 8.5c from Cochrane handbook version 5.0

Only Q5 & Q6 substantially differ from Cochrane criteria

http://www.cochrane-handbook.org/

Score as: 1 = adequate – described and appropriate – low risk of bias

0 = unclear – not described in sufficient detail – uncertain

-1 = obviously not done or described but not appropriate – high risk of bias

Q1 Randomisation process

Was the allocation sequence adequately generated?

| Examples that indicate YES - ADEQUATE | Examples that indicate NO - NOT DONE |
| --- | --- |
| The assignment of subjects to treatment gps is randomized, the sequence is generated externally - random number table, computer random number generator, coin tossing, shuffling cards, throwing dice, drawing lots | odd or even DOB, some rule based on date of admission, clinic record number, pt preference, Drs judgment, test results |

Q2 Allocation concealment

Was allocation adequately concealed?

| ADEQUATE | NOT DONE |
| --- | --- |
| central allocation - telephone, web, pharmacy; sequentially numbered sealed envelopes or identical drug containers | Investigators could forsee assignments and influence it - alternation, rotation, DOB, PID, other selection bias |

Q3 Blinding of outcome assessors

Who was blinded? – patient, health care prof, Data collector, Data analyst

Was knowledge of the allocated intervention adequately prevented during the study?

| ADEQUATE | NOT DONE |
| --- | --- |
| identical placebo for tablet studies, researcher and statistician for others, ipid results blinded, no blinding but lipid results (outcome measurement) not likely to be influenced by lack of blinding | no blinding and outcome measurement likely to be influenced – observer bias |

Q4 Incomplete outcome data

Were attrition and exclusions reported?

| ADEQUATE | NOT DONE |
| --- | --- |
| reasons for missing outcome data unlikely to be related to true outcome; missing outcome data balanced in numbers across groups with similar reasons; plausible effect size among missing outcomes not enough to have a clinically relevant impact on observed effect size, missing data imputed appropriately | not ITT analysis - as treated analysis done with substantial departure of intervention received from that assigned at randomization; imbalance in numbers or reasons for missing data across groups, no imputation or attrition data analysed to account for |

Q5 Selective Outcome reporting

Pre-specified primary outcomes reported

Power calculation done

| ADEQUATE | NOT APPROPRIATE |
| --- | --- |
| Primary outcomes pre-specified in protocol  Complete sample size recruited, as per power calculation | Not all outcomes have been reported; fails to report results for key outcome.  Study stopped prematurely |

Q6 Performance bias

Study limitations? Any confounding factors?

Was the study apparently free of other problems that could put it at a high risk of bias?

| ADEQUATE | NOT DONE |
| --- | --- |
| The treatment and control groups are similar at the start of the trial;  The only difference between groups is the treatment under investigation;  no other systematic differences in care provided  Measure of adherence for diet/tablets | extreme baseline imbalance; potential source of bias related to study design used e.g reviewed at diff time intervals, rec’d diff amount of attention from researchers; other confounding factors not accounted for (smoking, exercise, alcohol, use of PIs) |
