# Supplementary material for: The Effects of Dietary Intervention on HIV Dyslipidaemia: A Systematic Review and Meta-Analysis
Source: PLoS One. 2012 Jun 11;7(6):e38121. doi: 10.1371/journal.pone.0038121 (PMC3372478; doi:10.1371/journal.pone.0038121)
Supplement: Figure S2 — List of excluded studies and reasons. (DOC) [file pone.0038121.s002.doc]

**Supplementary Figure S2**

**List of excluded studies and reasons**

K. Abba, et al. 2008 intervention not to Rx dyslipidaemia

J. A. Aberg, et al. 2005 no diet intervention

A. Abubakar, et al. 2009 no diet intervention

M. Agostini, et al. 2009 diet not described, diet same in both arms, only exercise different

J. Amin, et al. 2003 no diet intervention

J. Ananworanich, et al. 2008 no diet intervention

S. Andrade, et al. 2002 no diet intervention

J. M. Baeten, et al. 2002 intervention not to Rx dyslipidaemia

J. M. Baeten, et al. 2007 no diet intervention

A. M. Bain, et al. 2008 wrong design

P. Barragan, et al. 2006 no diet intervention

A. Barrios, et al. 2002 no control group, found from ref list NOT Medline

M. K. Baum, et al. 2010 intervention not to Rx dyslipidaemia

B. M. Bergersen, et al. 2004 no diet intervention

M. Bickel, et al. 2006 no diet intervention

H. Buys, et al. 2002 wrong design

W. T. Cade, et al. 2010 diet same in both arms, only yoga different

P. E. Cahn, et al. 2004 no diet intervention

L. Calza, et al. 2002 no diet intervention

L. Calza, et al. 2003 no diet intervention

L. Calza, et al. 2009 no diet intervention

L. Calza, et al. 2005 no diet intervention

A. Campa and M. K. Baum 2010 wrong design

D. Carey, et al. 2010 no diet intervention

D. C. Chow, et al. 2010 no diet intervention

E. R. Christensen, et al. 2004 intervention not to Rx dyslipidaemia

J. Cofrancesco Jr, et al. 2009 wrong design

C. Cohen, et al. 2005 no diet intervention

B. Coll, et al. 2006 no diet intervention

J. D. Croxtall and C. M. Perry 2010 wrong design

F. De Lorenzo, et al. 2009 study protocol (no results)

C. De Simone, et al. 1994 intervention not for dyslipidaemia - carnitine

S. E. Dolan, et al. 2006 no diet, found from ref list NOT Medline

R. C. Dolman, et al. 2008 wrong design

M. L. Dreyfuss and W. W. Fawzi 2002 intervention not to Rx dyslipidaemia

J. J. Dugoua, et al. 2007 not HIV population

J. J. Eron, et al. 2010 no diet intervention

C. H. D. Fall, et al. 2003 intervention not to Rx dyslipidaemia

W. Fawzi 2003 intervention not to Rx dyslipidaemia

W. Fawzi, et al. 2005 intervention not to Rx dyslipidaemia

W. W. Fawzi and D. J. Hunter 1998 intervention not to Rx dyslipidaemia

W. W. Fawzi and G. I. Msamanga 1999 intervention not to Rx dyslipidaemia

W. W. Fawzi, et al. 2004 intervention not to Rx dyslipidaemia

C. J. Fichtenbaum 2004 wrong design

C. J. Fichtenbaum, et al. 2002 no diet intervention

T. Filippatos and H. J. Milionis 2008 not HIV population

C. Fisac, et al. 2005 no diet intervention

M. Fisher, et al. 2009 no diet intervention

M. Galli, et al. 2003 intervention not to Rx dyslipidaemia

M. Garland and W. W. Fawzi 1999 intervention not to Rx dyslipidaemia

C. L. Gibert, et al. 1999 intervention not to Rx dyslipidaemia

R. E. Gopane, et al. 2010 wrong design

P. Grant, et al. 2009 no diet intervention

M. L. Green 2002 no diet intervention

R. H. Haubrich, et al. 2009 no diet intervention

M. C. D. O. Izar 2007 wrong design

S. Jiamton, et al. 2003 intervention not to Rx dyslipidaemia

M. Johnson, et al. 2005 no diet intervention

P. Keiser and N. Nassar 2007 no diet intervention

P. H. Keiser, et al. 2005 no diet intervention

C. M. Kennedy, et al. 2000 wrong design

D. P. Kotler 1997 wrong design

P. Koutkia, et al. 2004 no diet intervention - Growth hormone

P. N. Kumar, et al. 2006 no diet intervention

R. Kupka, et al. 2008 intervention not to Rx dyslipidaemia

T. Lauritzen, et al. 1995 not HIV population

V. L. Leung and M. J. Glesby 2011 wrong design

P. Leyes, et al. 2008 wrong design - systematic review

E. Lioudaki, et al. 2011 wrong design

J. M. Llibre, et al. 2006 no diet intervention

S. Mahlungulu, et al. 2007 intervention not to Rx dyslipidaemia

P. W. G. Mallon, et al. 2006 no diet intervention

R. Manfredi and F. Chiodo 2001 no diet intervention

R. Manfredi, et al. 2005 not RCT

M. Markowitz, et al. 2005 no diet intervention

A. Martin, et al. 2004 no diet intervention

E. Martinez, et al. 2010 no diet intervention

M. Masia, et al. 2009 no diet intervention

G. McComsey, et al. 2003 no diet intervention

S. Mehta and W. Fawzi 2007 wrong design

S. Mehta, et al. 2010 wrong design

L. Michel, et al. 2010 wrong design

L. Milazzo, et al. 2009 no diet intervention

A. Milinkovic and J. Mallolas 2007 wrong design

J. Miller, et al. 2002 no diet intervention

J. Miller, et al. 2002 diet in both arms, only fibrate different

D. Mun, et al. 2008 HIV results mixed with non HIV

G. Moyle, et al. 2010 wrong design

G. J. Moyle, et al. 2001 no diet intervention

E. Mutimura, et al. 2008 no diet intervention

E. T. Overton and M. T. Yin 2011 wrong design

J. T. Parsons, et al. 2007 intervention not to Rx dyslipidaemia

C. Perez-Guzman, et al. 2005 not HIV population

C. Pichard, et al. 1998 intervention not to Rx dyslipidaemia

G. Pierone Jr, et al. 2006 no diet intervention

G. L. Plosker and L. J. Scott 2003 no diet intervention

D. Podzamczer, et al. 2007 no diet intervention

A. Poppa, et al. 2004 wrong design

L. M. Prescott and S. L. Prescott 2006 wrong design

E. Rabing, et al. 2004 treatment not for dyslipidaemia - coenz Q

A. P. Rahman, et al. 2008 no diet intervention

C. Reid and M. Courtney 2007 diet not described, wrong outcomes

C. Reid and M. Courtney 2007 lipids not reported

F. P. Robinson, et al. 2005 wrong design

K. Samaras, et al. 2010 not HIV population

S. L. Samson, et al. 2006 study protocol (no results)

R. B. Saper and R. Rash 2009 wrong design

F. R. Sattler, et al. 2008 intervention not to Rx dyslipidaemia

M. Schambelan, et al. 2002 wrong design

Y. K. Seedat, et al. 2006 wrong design

S. H. Sheth and R. J. Larson 2009 no diet intervention

G. K. Siberry, et al. 2002 wrong design

K. Squires, et al. 2004 no diet intervention

T. L. Stanley, et al. 2009 no diet intervention

J. T. Stapleton, et al. 2007 wrong design

C. A. Stone, et al. 2010 wrong design

M. Struwe, et al. 1993 intervention not to Rx dyslipidaemia

U. Suttmann, et al. 1996 intervention not to Rx dyslipidaemia

B. Swanson, et al. 2009 no diet intervention

R. Talwani, et al. 2002 wrong design

C. L. Taylor and E. G. L. Wilkins 2007 no diet intervention

P. Tebas 2007 wrong design

L. Terry, et al. 2006 diet same in both arms, only exercise different

J. S. Thompson Coon and E. Ernst 2003 not HIV population

P. C. Tien, et al. 2006 wrong design

M. Tungsiripat, et al. 2010 no diet intervention

A. Vasan, et al. 2006 no diet intervention

V. K. Vemuri, et al. 2008 no diet intervention

P. A. Venkatesh, et al. 2005 no diet intervention

E. Villamor, et al. 2005 intervention not to Rx dyslipidaemia

H. H. Vorster, et al. 2004 wrong design

C. A. Wanke, et al. 1996 intervention not to Rx dyslipidaemia

A. S. Wierzbicki, et al. 2008 wrong design

P. Williams, et al. 2009 wrong design - not RCT

Excluded references

1. Moyle GJ, Lloyd M, Reynolds B, Baldwin C, Mandalia S, Gazzard BG. Dietary advice with or without pravastatin for the management of hypercholesterolaemia associated with protease inhibitor therapy. *AIDS (London, England)* 2001: 1503-8.

2. Cade WT, Reeds DN, Mondy KE, et al. Yoga lifestyle intervention reduces blood pressure in HIV-infected adults with cardiovascular disease risk factors. *HIV Medicine* 2010; **11**: 379-88.

3. Agostini M, Lupo S, Palazzi J, Marconi L, Masante L. Systematized aerobic physical exercise and diet: Non-pharmacological treatment of lipodystrophy in HIV-positive patients on high-efficiency antiretroviral treatment [Spanish] Dieta y ejercicio fisico aerobico sistematizado: Tratamiento no farmacologico de la lipodistrofia en pacientes VIH positivos bajo tratamiento antiretroviral de alta eficacia. *Revista Medica de Rosario* 2009; **75**: 10-15.

4. De Simone C, Famularo G, Tzantzoglou S, Trinchieri V, Moretti S, Sorice F. Carnitine depletion in peripheral blood mononuclear cells from patients with AIDS: Effect of oral L-carnitine. *AIDS* 1994; **8**: 655-60.

5. Samaras K, Richardson R, Carr A. Postprandial lipid effects of low-dose ritonavir vs. raltegravir in HIV-uninfected adults. *AIDS* 2010; **24**: 1727-31.

6. Sattler FR, Rajicic N, Mulligan K, et al. Evaluation of high-protein supplementation in weight-stable HIV-positive subjects with a history of weight loss: A randomized, double-blind, multicenter trial. *Am J Clin Nutr* 2008; **88**: 1313-21.

7. Terry L, Sprinz E, Stein R, Medeiros NB, Oliveira J, Ribeiro JP. Exercise training in HIV-1-infected individuals with dyslipidemia and lipodystrophy. *Med Sci Sports Exerc* 2006; **38**: 411-17.

8. Leyes P, Martinez E, Forga M. Use of diet, nutritional supplements and exercise in HIV-infected patients receiving combination antiretroviral therapies: a systematic review. *Antivir Ther* 2008; **13**: 149-59.

9. Reid C, Courtney M. A randomized clinical trial to evaluate the effect of diet on weight loss and coping of people living with HIV and lipodystrophy. *Journal of Clinical Nursing* 2007; **16**: 197-206.

10. Rabing E, Stegger M, Jensen-Fangel S, Laursen AL, Ostergaard L. Mitochondrial DNA levels in fat and blood cells from patients with lipodystrophy or peripheral neuropathy and the effect of 90 days of high-dose coenzyme Q treatment: a randomized, double-blind, placebo-controlled pilot study. *Clin Infect Dis* 2004; **39**: 1371-9.

11. Williams P, Wu J, Cohn S, et al. Improvement in lipid profiles over 6 years of follow-up in adults with AIDS and immune reconstitution. *HIV Medicine* 2009; **10**: 290-301.

12. Barrios A, Blanco F, Garcia-Benayas T, et al. Effect of dietary intervention on highly active antiretroviral therapy-related dyslipemia. *AIDS* 2002; **16**: 2079-81.

13. Dolan SE, Frontera W, Librizzi J, et al. Effects of a supervised home-based aerobic and progressive resistance training regimen in women infected with human immunodeficiency virus: a randomized trial. *Arch Intern Med* 2006; **166**: 1225-31.

14. Miller J, Brown D, Amin J, et al. A randomized, double-blind study of gemfibrozil for the treatment of protease inhibitor-associated hypertriglyceridaemia. *AIDS* 2002; **16**: 2195-200.

15. Mun D, Engelson E, Albu J, Sharma M, Pitea T, Kotler D. Effects of Diet and Exercise and Rosiglitazone on Body Composition and Lipids, including Oxidized LDL in HIV+ and HIV- Men and Women. *Conference of Retrovirus and Opportunistic Infections* 2008: Abstract 944.

16. Manfredi R, Calza L, Chiodo F. Predominant hypertriglyceridemia caused by HAART administration: role of polyunsaturated omega-3 fatty acids, compared with the administration of fibrate derivatives. *3rd IAS Conference on HIV Pathogenesis and Treatment* 2005; **Rio de Janeiro**: Abstract TuPe2.2B03.

17. Reid C, Courtney M. A randomized clinical trial to evaluate the effect of diet on quality of life and mood of people living with HIV and lipodystrophy. *Journal of the Association of Nurses in AIDS Care* 2007; **18**: 3-11.

18. Engelson E, Agin D, Kenya S, et al. The effects of a diet and exercise weight loss program in obese HIV-infected women XIV International AIDS Conference; 2002.

19. Abba K, Sudarsanam TD, Grobler L, Volmink J. Nutritional supplements for people being treated for active tuberculosis. *Cochrane Database of Systematic Reviews* 2008.

20. Aberg JA, Zackin RA, Brobst SW, et al. A randomized trial of the efficacy and safety of fenofibrate versus pravastatin in HIV-infected subjects with lipid abnormalities: AIDS Clinical Trials Group study 5087. *AIDS Research and Human Retroviruses* 2005; **21**: 757-67.

21. Abubakar A, Holding P, Newton CRJC, Van Baar A, Van De Vijver FJR. The role of weight for age and disease stage in poor psychomotor outcome of HIV-infected children in Kilifi, Kenya. *Developmental Medicine and Child Neurology* 2009; **51**: 968-73.

22. Amin J, Moore A, Carr A, et al. Combined analysis of two-year follow-up from two open-label randomized trials comparing efficacy of three nucleoside reverse transcriptase inhibitor backbones for previously untreated HIV-1 infection: Ozcombo 1 and 2. *HIV Clinical Trials* 2003; **4**: 252-61.

23. Ananworanich J, Nuesch R, Cote HCF, et al. Changes in metabolic toxicity after switching from stavudine/didanosine to tenofovir/lamivudine - A Staccato trial substudy. *Journal of Antimicrobial Chemotherapy* 2008; **61**: 1340-43.

24. Andrade S, Lan SJJ, Engelson ES, et al. Use of a Durnin-Womersley formula to estimate change in subcutaneous fat content in HIV-infected subjects. *American Journal of Clinical Nutrition* 2002; **75**: 587-92.

25. Baeten JM, McClelland RS, Richardson BA, et al. Vitamin A deficiency and the acute phase response among HIV-1-infected and -uninfected women in Kenya. *Journal of Acquired Immune Deficiency Syndromes* 2002; **31**: 243-49.

26. Baeten JM, McClelland RS, Wener MH, et al. Relationship between markers of HIV-1 disease progression and serum beta-carotene concentrations in Kenyan women. *International Journal of STD and AIDS* 2007; **18**: 202-06.

27. Bain AM, White EA, Rutherford WS, Rahman AP, Busti AJ. A multimodal, evidence-based approach to achieve lipid targets in the treatment of antiretroviral-associated dyslipidemia: Case report and review of the literature. *Pharmacotherapy* 2008; **28**: 932-38.

28. Barragan P, Fisac C, Podzamczer D. Switching strategies to improve lipid profile and morphologic changes. *AIDS Reviews* 2006; **8**: 191-203.

29. Baum MK, Lai S, Sales S, Page JB, Campa A. Randomized, controlled clinical trial of zinc supplementation to prevent immunological failure in HIV-infected adults. *Clinical Infectious Diseases* 2010; **50**: 1653-60.

30. Bergersen BM, Sandvik L, Bruun JN, Tonstad S. Elevated Framingham risk in HIV-positive on highly active antiretroviral therapy: Results from a Norwegian study of 721 subjects. *European Journal of Clinical Microbiology and Infectious Diseases* 2004; **23**: 625-30.

31. Bickel M, Zangos S, Jacobi V, et al. A randomized, open-label study to compare the effects of two different doses of recombinant human growth hormone on fat reduction and fasting metabolic parameters in HIV-1-infected patients with lipodystrophy. *HIV Medicine* 2006; **7**: 397-403.

32. Buys H, Hendricks M, Eley B, Hussey G. The role of nutrition and micronutrients in paediatric HIV infection. *SADJ : journal of the South African Dental Association = tydskrif van die Suid-Afrikaanse Tandheelkundige Vereniging* 2002; **57**: 454-56.

33. Cahn PE, Gatell JM, Squires K, et al. Atazanavir--a once-daily HIV protease inhibitor that does not cause dyslipidemia in newly treated patients: results from two randomized clinical trials. *Journal of the International Association of Physicians in AIDS Care (Chicago, Ill : 2002)* 2004; **3**: 92-98.

34. Calza L, Manfredi R, Chiodo F. Use of fibrates in the management of hyperlipidemia in HIV-infected patients receiving HAART. *Infection* 2002; **30**: 26-31.

35. Calza L, Manfredi R, Chiodo F. Statins and fibrates for the treatment of hyperlipidaemia in HIV-infected patients receiving HAART. *AIDS* 2003; **17**: 851-59.

36. Calza L, Manfredi R, Colangeli V, et al. Efficacy and safety of atazanavir-ritonavir plus abacavir-lamivudine or tenofovir-emtricitabine in patients with hyperlipidaemia switched from a stable protease inhibitor-based regimen including one thymidine analogue. *AIDS Patient Care and STDs* 2009; **23**: 691-97.

37. Calza L, Manfredi R, Colangeli V, et al. Substitution of nevirapine or efavirenz for protease inhibitor versus lipid-lowering therapy for the management of dyslipidaemia. *AIDS* 2005; **19**: 1051-58.

38. Campa A, Baum MK. Micronutrients and HIV infection. *HIV Therapy* 2010; **4**: 437-69.

39. Carey D, Amin J, Boyd M, Petoumenos K, Emery S. Lipid profiles in HIV-infected adults receiving atazanavir and atazanavir/ritonavir: Systematic review and meta-analysis of randomized controlled trials. *Journal of Antimicrobial Chemotherapy* 2010; **65**: 1878-88.

40. Chow DC, Stein JH, Seto TB, et al. Short-term effects of extended-release niacin on endothelial function in HIV-infected patients on stable antiretroviral therapy. *AIDS* 2010; **24**: 1019-23.

41. Christensen ER, Stegger M, Jensen-Fangel S, Laursen AL, Ostergaard L. Mitochondrial DNA levels in fat and blood cells from patients with lipodystrophy or peripheral neuropathy and the effect of 90 days of high-dose coenzyme Q treatment: A randomized, double-blind, placebo-controlled pilot study. *Clinical Infectious Diseases* 2004; **39**: 1371-79.

42. Cofrancesco Jr J, Freedland E, McComsey G. Treatment options for HIV-associated central fat accumulation. *AIDS Patient Care and STDs* 2009; **23**: 5-18.

43. Cohen C, Nieto-Cisneros L, Zala C, et al. Comparison of atazanavir with lopinavir/ritonavir in patients with prior protease inhibitor failure: A randomized multinational trial. *Current Medical Research and Opinion* 2005; **21**: 1683-92.

44. Coll B, van Wijk JPH, Parra S, et al. Effects of rosiglitazone and metformin on postprandial paraoxonase-1 and monocyte chemoattractant protein-1 in human immunodeficiency virus-infected patients with lipodystrophy. *European Journal of Pharmacology* 2006; **544**: 104-10.

45. Croxtall JD, Perry CM. LopinavirRitonavir: A review of its use in the management of HIV-1 infection. *Drugs* 2010; **70**: 1885-915.

46. De Lorenzo F, Boffito M, Collot-Teixeira S, et al. Prevention of atherosclerosis in patients living with HIV.

47. De Lorenzo F, Boffito M, Collot-Teixeira S, et al. Prevention of atherosclerosis in patients living with HIV. *Vascular Health and Risk Management* 2009; **5**: 287-300.

48. Dolman RC, Stonehouse W, Riet HV, Badham J, Jerling JC. Beliefs of South Africans regarding food and cardiovascular health. *Public Health Nutrition* 2008; **11**: 946-54.

49. Dreyfuss ML, Fawzi WW. Micronutrients and vertical transmission of HIV-1. *American Journal of Clinical Nutrition* 2002; **75**: 959-70.

50. Dugoua JJ, Seely D, Perri D, et al. From type 2 diabetes to antioxidant activity: A systematic review of the safety and efficacy of common and cassia cinnamon bark. *Canadian Journal of Physiology and Pharmacology* 2007; **85**: 837-47.

51. Eron JJ, Young B, Cooper DA, et al. Switch to a raltegravir-based regimen versus continuation of a lopinavir-ritonavir-based regimen in stable HIV-infected patients with suppressed viraemia (SWITCHMRK 1 and 2): two multicentre, double-blind, randomised controlled trials. *The Lancet* 2010; **375**: 396-407.

52. Fall CHD, Yajnik CS, Rao S, Davies AA, Brown N, Farrant HJW. Micronutrients and fetal growth. *Journal of Nutrition* 2003; **133**: 1747S-56S.

53. Fawzi W. Micronutrients and human immunodeficiency virus type 1 disease progression among adults and children. *Clinical Infectious Diseases* 2003; **37**: S112-S16.

54. Fawzi W, Msamanga G, Spiegelman D, Hunter DJ. Studies of vitamins and minerals and HIV transmission and disease progression. *Journal of Nutrition* 2005; **135**: 938-44.

55. Fawzi WW, Hunter DJ. Vitamins in HIV disease progression and vertical transmission. *Epidemiology* 1998; **9**: 457-66.

56. Fawzi WW, Msamanga GI. Epidemiological studies of vitamins among HIV-infected women and children. *Prenatal and Neonatal Medicine* 1999; **4**: 386-90.

57. Fawzi WW, Msamanga GI, Spiegelman D, et al. A randomized trial of multivitamin supplements and HIV disease progression and mortality. *New England Journal of Medicine* 2004; **351**: 23-32.

58. Fichtenbaum CJ. Coronary heart disease risk, dyslipidemia, and management in HIV-infected persons. *HIV Clinical Trials* 2004; **5**: 416-33.

59. Fichtenbaum CJ, Gerber JG, Rosenkranz SL, et al. Pharmacokinetic interactions between protease inhibitors and statins in HIV seronegative volunteers: ACTG study A5047. *AIDS* 2002; **16**: 569-77.

60. Filippatos T, Milionis HJ. Treatment of hyperlipidaemia with Fenofibrate and related fibrates. *Expert Opinion on Investigational Drugs* 2008; **17**: 1599-614.

61. Fisac C, Fumero E, Crespo M, et al. Metabolic benefits 24 months after replacing a protease inhibitor with abacavir, efavirenz or nevirapine. *AIDS* 2005; **19**: 917-25.

62. Fisher M, Moyle GJ, Shahmanesh M, et al. A randomized comparative trial of continued zidovudine/lamivudine or replacement with tenofovir disoproxil fumarate/emtricitabine in efavirenz-treated HIV-1-infected individuals. *Journal of Acquired Immune Deficiency Syndromes* 2009; **51**: 562-68.

63. Galli M, Gervasoni C, Ridolfo AL, et al. Cytokine production in women with antiretroviral treatment-associated breast fat accumulation and limb wasting. *AIDS* 2003; **17**: S155-S61.

64. Garland M, Fawzi WW. Antioxidants and progression of human immunodeficiency virus (HIV) disease. *Nutrition Research* 1999; **19**: 1259-76.

65. Gibert CL, Wheeler DA, Collins G, et al. Randomized, controlled trial of caloric supplements in HIV infection. *Journal of Acquired Immune Deficiency Syndromes and Human Retrovirology* 1999; **22**: 253-59.

66. Gopane RE, Pisa PT, Vorster HH, Kruger A, Margetts BM. Relationships of alcohol intake with biological health outcomes in an African population in transition: The Transition and Health during Urbanisation in South Africa (THUSA) study. *South African Journal of Clinical Nutrition* 2010; **23**: S16-S21.

67. Grant P, Taylor J, Cain P, et al. Maintaining reduced viral fitness and CD4 response in HIV-infected patients with viremia receiving a boosted protease inhibitor. *Clinical Infectious Diseases* 2009; **48**: 680-82.

68. Green ML. Evaluation and management of dyslipidemia in patients with HIV infection. *Journal of General Internal Medicine* 2002; **17**: 797-810.

69. Haubrich RH, Riddler SA, DiRienzo AG, et al. Metabolic outcomes in a randomized trial of nucleoside, nonnucleoside and protease inhibitor-sparing regimens for initial HIV treatment. *AIDS* 2009; **23**: 1109-18.

70. Izar MCDO. Dyslipidemia [Portuguese] Dislipidemias. *Revista Brasileira de Medicina* 2007; **64**: 140-60.

71. Jiamton S, Pepin J, Suttent R, et al. A randomized trial of the impact of multiple micronutrient supplementation on mortality among HIV-infected individuals living in Bangkok. *AIDS* 2003; **17**: 2461-69.

72. Johnson M, Grinsztejn B, Rodriguez C, et al. Atazanavir plus ritonavir or saquinavir, and lopinavir/ritonavir in patients experiencing multiple virological failures. *AIDS* 2005; **19**: 685-94.

73. Keiser P, Nassar N. Abacavir sulfate/lamivudine/zidovudine fixed combination in the treatment of HIV infection. *Expert Opinion on Pharmacotherapy* 2007; **8**: 477-83.

74. Keiser PH, Sension MG, DeJesus E, et al. Substituting abacavir for hyperlipidemia-associated protease inhibitors in HAART regimens improves fasting lipid profiles, maintains virologic suppression, and simplifies treatment. *BMC Infectious Diseases* 2005; **5**.

75. Kennedy CM, Kuhn L, Stein Z. Vitamin A and HIV infection: Disease progression, mortality, and transmission. *Nutrition Reviews* 2000; **58**: 291-303.

76. Kotler DP. Management of nutritional alterations and issues concerning quality of life. *Journal of Acquired Immune Deficiency Syndromes and Human Retrovirology* 1997; **16**: S30-S35.

77. Koutkia P, Canavan B, Breu J, Torriani M, Kissko J, Grinspoon S. Growth hormone - Releasing hormone in HIV-infected men with lipodystrophy: A randomized controlled trial. *Journal of the American Medical Association* 2004; **292**: 210-18.

78. Kumar PN, Rodriguez-French A, Thompson MA, et al. A prospective, 96-week study of the impact of Trizivir, Combivir/nelfinavir, and lamivudine/stavudine/nelfinavir on lipids, metabolic parameters and efficacy in antiretroviral-naive patients: Effect of sex and ethnicity. *HIV Medicine* 2006; **7**: 85-98.

79. Kupka R, Mugusi F, Aboud S, et al. Randomized, double-blind, placebo-controlled trial of selenium supplements among HIV-infected pregnant women in Tanzania: Effects on maternal and child outcomes. *American Journal of Clinical Nutrition* 2008; **87**: 1802-08.

80. Lauritzen T, Leboeuf-Yde C, Lunde IM, Nielsen KDB. Ebeltoft project: Baseline data from a five-year randomized, controlled, prospective health promotion study in a Danish population. *British Journal of General Practice* 1995; **45**: 542-47.

81. Leung VL, Glesby MJ. Pathogenesis and treatment of HIV lipohypertrophy. *Current Opinion in Infectious Diseases* 2011; **24**: 43-49.

82. Lioudaki E, Ganotakis ES, Mikhailidis DP. Ezetimibe; more than a low density lipoprotein cholesterol lowering drug? an update after 4 years. *Current Vascular Pharmacology* 2011; **9**: 62-86.

83. Llibre JM, Domingo P, Palacios R, et al. Sustained improvement of dyslipidaemia in HAART-treated patients replacing stavudine with tenofovir. *AIDS* 2006; **20**: 1407-14.

84. Mahlungulu S, Grobler LA, Visser ME, Volmink J. Nutritional interventions for reducing morbidity and mortality in people with HIV. *Cochrane database of systematic reviews (Online)* 2007.

85. Mallon PWG, Miller J, Kovacic JC, et al. Effect of pravastatin on body composition and markers of cardiovascular disease in HIV-infected men - A randomized, placebo-controlled study. *AIDS* 2006; **20**: 1003-10.

86. Manfredi R, Chiodo F. Disorders of lipid metabolism in patients with HIV disease treated with antiretroviral agents: Frequency, relationship with administered drugs, and role of hypolipidaemic therapy with bezafibrate. *Journal of Infection* 2001; **42**: 181-88.

87. Markowitz M, Hill-Zabala C, Lang J, et al. Induction with abacavir/lamivudine/zidovudine plus efavirenz for 48 weeks followed by 48-week maintenance with abacavir/lamivudine/zidovudine alone in antiretroviral-naive HIV-1-infected patients. *Journal of Acquired Immune Deficiency Syndromes* 2005; **39**: 257-64.

88. Martin A, Smith D, Carr A, et al. Progression of lipodystrophy (LD) with continued thymidine analogue usage: Long-term follow-up from a randomized clinical trial (the PIILR study). *HIV Clinical Trials* 2004; **5**: 192-200.

89. Martin A, Smith DE, Carr A, et al. Reversibility of lipoatrophy in HIV-infected patients 2 years after switching from a thymidine analogue to abacavir: The MITOX Extension Study. *AIDS* 2004; **18**: 1029-36.

90. Martinez E, Larrousse M, Llibre JM, et al. Substitution of raltegravir for ritonavir-boosted protease inhibitors in HIV-infected patients: the SPIRAL study. *AIDS* 2010; **24**: 1697-707.

91. Masia M, Bernal E, Padilla S, et al. A pilot randomized trial comparing an intensive versus a standard intervention in stable HIV-infected patients with moderate - High cardiovascular risk. *Journal of Antimicrobial Chemotherapy* 2009; **64**: 589-98.

92. McComsey G, Bhumbra N, Ma JF, Rathore M, Alvarez A. Impact of protease inhibitor substitution with efavirenz in HIV-infected children: results of the First Pediatric Switch Study. *Pediatrics* 2003; **111**: e275-81.

93. Mehta S, Fawzi W. Effects of Vitamins, Including Vitamin A, on HIV/AIDS Patients. *Vitamin A* 2007; **75**: 355-83.

94. Mehta S, Giovannucci E, Mugusi FM, et al. Vitamin D status of HIV-infected women and its association with hiv disease progression, anemia, and mortality. *PLoS ONE* 2010; **5**.

95. Michel L, Carrieri MP, Fugon L, et al. Harmful alcohol consumption and patterns of substance use in HIV-infected patients receiving antiretrovirals (ANRS-EN12-VESPA Study): Relevance for clinical management and intervention. *AIDS Care - Psychological and Socio-Medical Aspects of AIDS/HIV* 2010; **22**: 1136-45.

96. Milazzo L, Meroni L, Galazzi M, et al. Does fluvastatin favour HCV replication in vivo? A pilot study on HIV-HCV coinfected patients. *Journal of Viral Hepatitis* 2009; **16**: 479-84.

97. Milinkovic A, Mallolas J. Fixed-dose combination of abacavir, lamivudine and zidovudine for HIV therapy. *Future Virology* 2007; **2**: 23-30.

98. Moyle G, Moutschen M, Martinez E, et al. Epidemiology, assessment, and management of excess abdominal fat in persons with HIV infection. *AIDS Reviews* 2010; **12**: 3-14.

99. Moyle GJ, Lloyd M, Reynolds B, Baldwin C, Mandalia S, Gazzard BG. Dietary advice with or without pravastatin for the management of hypercholesterolaemia associated with protease inhibitor therapy. *AIDS* 2001; **15**: 1503-08.

100. Mutimura E, Crowther NJ, Cade TW, Yarasheski KE, Stewart A. Exercise training reduces central adiposity and improves metabolic indices in HAART-treated HIV-positive subjects in Rwanda: A randomized controlled trial. *AIDS Res Hum Retroviruses* 2008; **24**: 15-23.

101. Mutimura E, Stewart A, Crowther NJ, Yarasheski KE, Cade WT. The effects of exercise training on quality of life in HAART-treated HIV-positive Rwandan subjects with body fat redistribution. *Quality of Life Research* 2008; **17**: 377-85.

102. Overton ET, Yin MT. The rapidly evolving research on vitamin D among HIV-infected populations. *Current Infectious Disease Reports* 2011; **13**: 83-93.

103. Parsons JT, Golub SA, Rosof E, Holder C. Motivational interviewing and cognitive-behavioral intervention to improve HIV medication adherence among hazardous drinkers: A randomized controlled trial. *Journal of Acquired Immune Deficiency Syndromes* 2007; **46**: 443-50.

104. Perez-Guzman C, Vargas MH, Quinonez F, Bazavilvazo N, Aguilar A. A cholesterol-rich diet accelerates bacteriologic sterilization in pulmonary tuberculosis. *Chest* 2005; **127**: 643-51.

105. Pichard C, Sudre P, Karsegard V, et al. A randomized double-blind controlled study of 6 months of oral nutritional supplementation with arginine and -3 fatty acids in HIV-infected patients. *AIDS* 1998; **12**: 53-63.

106. Pierone Jr G, Mieras J, Bulgin-Coleman D, et al. A pilot study of switch to lopinavir/ritonavir (LPV/r) monotherapy from nonnucleoside reverse transcriptase inhibitor-based therapy. *HIV Clinical Trials* 2006; **7**: 237-45.

107. Plosker GL, Scott LJ. Saquinavir: A review of its use in boosted regimens for treating HIV infection. *Drugs* 2003; **63**: 1299-324.

108. Podzamczer D, Ferrer E, Sanchez P, et al. Less lipoatrophy and better lipid profile with abacavir as compared to stavudine: 96-Week results of a randomized study. *Journal of Acquired Immune Deficiency Syndromes* 2007; **44**: 139-47.

109. Poppa A, Davidson O, Deutsch J, et al. British HIV Association(BHIVA)/British Association for Sexual Health and HIV (BASHH) guidelines on provision of adherence support to individuals receiving antiretroviral therapy (2003). *HIV Medicine* 2004; **5**: 46-60.

110. Prescott LM, Prescott SL. American Heart Association Scientific Session 2005. *P and T* 2006; **31**: 48-51.

111. Rahman AP, Eaton SA, Nguyen ST, et al. Safety and efficacy of simvastatin for the treatment of dyslipidemia in human immunodeficiency virus-infected patients receiving efavirenz-based highly active antiretroviral therapy. *Pharmacotherapy* 2008; **28**: 913-19.

112. Robinson FP, Hoff JA, Kondos GT. Coronary artery calcium in HIV-infected men treated with highly active antiretroviral therapy. *The Journal of cardiovascular nursing* 2005; **20**: 149-54.

113. Samson SL, Pownall HJ, Scott LW, et al. Heart positive: Design of a randomized controlled clinical trial of intensive lifestyle intervention, niacin and fenofibrate for HIV lipodystrophy/dyslipidemia. *Contemporary Clinical Trials* 2006; **27**: 518-30.

114. Saper RB, Rash R. Zinc: An essential micronutrient. *American Family Physician* 2009; **79**: 768-72.

115. Schambelan M, Benson CA, Carr A, et al. Management of metabolic complications associated with antiretroviral therapy for HIV-1 infection: Recommendations of an International AIDS Society-USA Panel. *Journal of Acquired Immune Deficiency Syndromes* 2002; **31**: 257-75.

116. Seedat YK, Croasdale MA, Milne FJ, et al. South african hypertension guideline 2006. *South African Medical Journal* 2006; **96**: 337-62.

117. Sheth SH, Larson RJ. The efficacy and safety of insulin-sensitizing drugs in HIV-associated lipodystrophy syndrome: A meta-analysis of randomized trials. *BMC Infectious Diseases* 2009; **10**.

118. Siberry GK, Ruff AJ, Black R. Zinc and human immunodeficiency virus infection. *Nutrition Research* 2002; **22**: 527-38.

119. Squires K, Lazzarin A, Gatell JM, et al. Comparison of once-daily atazanavir with efavirenz, each in combination with fixed-dose zidovudine and lamivudine, as initial therapy for patients infected with HIV. *Journal of Acquired Immune Deficiency Syndromes* 2004; **36**: 1011-19.

120. Stanley TL, Joy T, Hadigan CM, et al. Effects of switching from lopinavir/ritonavir to atazanavir/ritonavir on muscle glucose uptake and visceral fat in HIV-infected patients. *AIDS* 2009; **23**: 1349-57.

121. Stapleton JT, Bennett K, Bosch RJ, Polgreen PM, Swindells S. Effect of antiretroviral therapy and hepatitis C co-infection on changes in lipid levels in HIV-infected patients 48 weeks after initiation of therapy. *HIV Clinical Trials* 2007; **8**: 429-36.

122. Stone CA, Kawai K, Kupka R, Fawzi WW. Role of selenium in HIV infection. *Nutrition Reviews* 2010; **68**: 671-81.

123. Struwe M, Kaempfer SH, Geiger CJ, et al. Effect of dronabinol on nutritional status in HIV infection. *Annals of Pharmacotherapy* 1993; **27**: 827-31.

124. Suttmann U, Ockenga J, Schneider H, et al. Weight gain and increased concentrations of receptor proteins for tumor necrosis factor after patients with symptomatic HIV infection received fortified nutrition support. *Journal of the American Dietetic Association* 1996; **96**: 565-69.

125. Swanson B, Sha BE, Keithley JK, et al. Lipoprotein particle profiles by nuclear magnetic resonance spectroscopy in medically underserved HIV-infected persons. *Journal of Clinical Lipidology* 2009; **3**: 379-84.

126. Talwani R, Falusi OM, Mendes de Leon CF, et al. Electron beam computed tomography for assessment of coronary artery disease in HIV-infected men receiving antiretroviral therapy. *Journal of Acquired Immune Deficiency Syndromes* 2002; **30**: 191-95.

127. Taylor CL, Wilkins EGL. Abacavir/lamivudine fixed-dose combination tablet for the treatment of HIV-1 infection. *Future Virology* 2007; **2**: 11-21.

128. Tebas P. Interventions for body habitus changes associated with HIV infection and its treatment. *Current HIV/AIDS Reports* 2007; **4**: 86-92.

129. Thompson Coon JS, Ernst E. Herbs for serum cholesterol reduction: A systematic review. *Journal of Family Practice* 2003; **52**: 468-78.

130. Tien PC, Benson C, Zolopa AR, Sidney S, Osmond D, Grunfeld C. The Study of Fat Redistribution and Metabolic Change in HIV Infection (FRAM): Methods, design, and sample characteristics. *American Journal of Epidemiology* 2006; **163**: 860-69.

131. Tungsiripat M, Bejjani DE, Rizk N, et al. Rosiglitazone improves lipoatrophy in patients receiving thymidine-sparing regimens. *AIDS* 2010; **24**: 1291-98.

132. Tungsiripat M, Kitch D, Glesby MJ, et al. A pilot study to determine the impact on dyslipidemia of adding tenofovir to stable background antiretroviral therapy: ACTG 5206. *AIDS* 2010; **24**: 1781-4.

133. Vasan A, Renjifo B, Hertzmark E, et al. Different rates of disease progression of HIV type 1 infection in Tanzania based on infecting subtype. *Clinical Infectious Diseases* 2006; **42**: 843-52.

134. Vemuri VK, Janero DR, Makriyannis A. Pharmacotherapeutic targeting of the endocannabinoid signaling system: Drugs for obesity and the metabolic syndrome. *Physiology and Behavior* 2008; **93**: 671-86.

135. Venkatesh PA, Bosch RJ, McIntosh K, Mugusi F, Msamanga G, Fawzi WW. Predictors of incident tuberculosis among HIV-1-infected women in Tanzania. *International Journal of Tuberculosis and Lung Disease* 2005; **9**: 1105-11.

136. Villamor E, Saathoff E, Manji K, Msamanga G, Hunter DJ, Fawzi WW. Vitamin supplements, socioeconomic status, and morbidity events as predictors of wasting in HIV-infected women from Tanzania. *The American journal of clinical nutrition* 2005; **82**: 857-65.

137. Vorster HH, Kruger A, Margetts BM, et al. The nutritional status of asymptomatic HIV-infected Africans: Directions for dietary intervention? *Public Health Nutrition* 2004; **7**: 1055-64.

138. Wanke CA, Pleskow D, Degirolami PC, Lambl BB, Merkel K, Akrabawi S. A medium chain triglyceride-based diet in patients with HIV and chronic diarrhea reduces diarrhea and malabsorption: a prospective, controlled trial. *Nutrition* 1996; **12**: 766-71.

139. Wierzbicki AS, Purdon SD, Hardman TC, Kulasegaram R, Peters BS. HIV lipodystrophy and its metabolic consequences: Implications for clinical practice. *Current Medical Research and Opinion* 2008; **24**: 609-24.
