# Supplementary material for: The Effects of Dietary Intervention on HIV Dyslipidaemia: A Systematic Review and Meta-Analysis
Source: PLoS One. 2012 Jun 11;7(6):e38121. doi: 10.1371/journal.pone.0038121 (PMC3372478; doi:10.1371/journal.pone.0038121)
Supplement: Figure S4 — Adherence to diet – Graphs to show levels of nutrients advised and consumed. (DOC) [file pone.0038121.s004.doc]

Supplementary Figure **S4**

**Adherence to diet – Graphs to show levels of nutrients advised and consumed**
