# Supplementary material for: The Effects of Dietary Intervention on HIV Dyslipidaemia: A Systematic Review and Meta-Analysis
Source: PLoS One. 2012 Jun 11;7(6):e38121. doi: 10.1371/journal.pone.0038121 (PMC3372478; doi:10.1371/journal.pone.0038121)
Supplement: Table S1 — Search strategy. (DOC) [file pone.0038121.s007.doc]

**Table S1** Search strategy

|  | Freetext | MeSH |
| --- | --- | --- |
| Population | HIV* | Exp HIV INFECTIONS/ |
| Intervention | diet* adj therap*  diet* adj intervention*  diet* adj treatment*  diet* adj3 plan  diet* adj3 change*  lifestyle adj3 change*  lifestyle adj3 program*  lipid* adj3 modifi*  lipid* adj3 low*  lipid* adj3 reduc*  fat* adj3 low*  fat* adj3 modif*  fat* adj3 saturat  soluble adj fib?r  viscous adj fib?r  wholegrain adj cereal*  legume*  bean*  plant adj stanol*  Mediterranean adj diet  Portfolio adj diet  Cardioprotective adj diet  Medical nutrition therapy  Diet, reducing – already in diet therapy | exp DIET THERAPY/ OR exp DIET/ OR FOOD HABITS/ OR exp LIFESTYLE/  exp DIET ATHEROGENIC/  exp AVENA SATIVA/ OR exp HORDEUM/ OR exp DIETARY FIBER/ OR exp NUTS/ OR exp FRUIT/ OR exp VEGETABLES/ OR exp FATS/ OR exp OILS/ OR exp OLEA  PHYTOSTEROLS/  SOYBEAN PROTEIN/  FABACEAE/ |
| Outcome | Heart attack  Cardiovascular*  Cholesterol*  Triglycerid*  Lipoprotein*  LDL-cholesterol OR VLDL-cholesterol OR HDL-cholesterol  Dyslipid* OR Hypercholestrol* OR Hyperlipid* OR Hypertriglycerid*  Disease adj progression | exp LIPID METABOLISM DISORDERS/ OR  exp MYOCARDIAL ISCHEMIA/ |
| Study Design | Publication types: RCT + controlled clinical trial | Limit to humans |
