# Supplementary material for: The Effects of Dietary Intervention on HIV Dyslipidaemia: A Systematic Review and Meta-Analysis
Source: PLoS One. 2012 Jun 11;7(6):e38121. doi: 10.1371/journal.pone.0038121 (PMC3372478; doi:10.1371/journal.pone.0038121)
Supplement: Table S2 — Fish oil doses. (DOC) [file pone.0038121.s008.doc]

**Supplementary Table S2**

Fish oil doses

| STUDY | INTERVENTION | DOSE EPA | DOSE DHA | EPA + DHA mg | DOSE fish oil |
| --- | --- | --- | --- | --- | --- |
| Thusgaard 2009 | 2 Omacor bd (4x 460mg EPA + 380mg DHA +4mg vit E) | 1840 | 1520  +16mg vit E (as d-alpha tocopherol) | 3360 | 3.6g |
| Carter 2006 | 3 Maxepa tds 9g (9x EPA 180mg + DHA 120mg) + diet | 1620 | 1080 | 2700 | 9g |
| Baril 2007 | 1g salmon oil tds (3x180mgEPA+120mgDHA) | 540 | 360 | 900 | 3g |
| Gerber 2008 | 3g fish oil bd (6x 500mg EPA +310mg DHA +190mg other omega 3 + 20IU d-alpha tocopherol) | 3000 | 1860  +120IU vit E (80mg) | 4860 | 6g |
| Wohl 2005 | Fish oil (1750mg EPA + 1150mg DHA) +diet + exercise | 1750 | 1150  + 15IU vit E (10mg) (as d-alpha tocopherol) | 2900 | 10g |
| DeTruchis 2007 | 2g n-3PUFA tds (Maxepa 18%EPA, 12% DHA) +diet | 1080 | 720 | 1800 | 6g |
| Woods 2009 | 5 x capsules/d (Omega Rx 400mg EPA + 200mg DHA)  control gp intake from diet at wk 13 =173mg | 2000 | 1000 | 3000 from supplements + 3000 from diet  (actual intake 4723mg at wk 13) | See table 2 EPA intake & DHA intake error with units |
| Peters 2012 | 2 Omacor bd | 1840 | 1520 | 3360 | 3.6g |
| Peabody 2002 |  |  |  |  | 300mg |
