# Supplementary material for: The Effects of Dietary Intervention on HIV Dyslipidaemia: A Systematic Review and Meta-Analysis
Source: PLoS One. 2012 Jun 11;7(6):e38121. doi: 10.1371/journal.pone.0038121 (PMC3372478; doi:10.1371/journal.pone.0038121)
Supplement: Table S3 — Dietary interventions. (DOC) [file pone.0038121.s009.doc]

**Table S3**

| Ref | Fat | Sat fat | PUFA & MUFA | Omega 3 | Fibre | CHO | Other | Modelled on | Dietary intake attained by intervention group |
| --- | --- | --- | --- | --- | --- | --- | --- | --- | --- |
| Fitch | <35% of calories | <7% of calories | Up to 10% of calories PUFA; up to 20% of calories MUFA | Emphasis on sources | 25-35g/d |  | Exercise 3hr/wk = 10,000 steps/d | NCEPIII, AACE, DPP | -347kcal/d; sat fat 10% of calories; 17g fibre |
| Woods | ~25% of calories | ~7% of calories | 1:1:1 of sat:PUFA:MUFA | 3g/d n-3 fatty acid diet + 3g/d supplements | >40g/d | Low GI | 100mg/d Vit E, energy Rx for weight stable |  | 2306kcal; fat 39%, sat fat 9%, PUFA 11%, MUFA 11% of calories; 37g fibre |
| Sanchez |  | <7% | PUFA reduced, cholesterol <200mg/d | Emphasis on sources, or sesame and linen grains or oatbran | 20g/d | Low GI; alcohol reduced | Exercise 3hr/week – 20 mins aerobic + PRE all muscle gps | NCEPIII | Actual intake not reported |
| Lazzaretti | ~25% of calories | ~5% of calories | 10% of calories from MUFA; 10% of calories from PUFA; <200mg/d cholesterol |  | 30g/d | Reduced sugar | Energy Rx to reach and maintain appropriate weight | NCEPIII | Reported graphically,  approx results: fat 20%, sat fat 20%, PUFA 5%, MUFA 8% of calories; 33g fibre |

**Dietary interventions**

Sat fat saturated fat; MUFA monounsaturated fatty acids; PUFA polyunsaturated fatty acids;

CHO carbohydrate; GI glycaemic index;

NCEPIII National Cholesterol Education Program Adult Treatment Panel III Guidelines;

American Association of Clinical Endocrinologists; Diabetes Prevention Program;

Rx treatment; PRE progressive resistance exercise
