# Supplementary material for: The Effects of Dietary Intervention on HIV Dyslipidaemia: A Systematic Review and Meta-Analysis
Source: PLoS One. 2012 Jun 11;7(6):e38121. doi: 10.1371/journal.pone.0038121 (PMC3372478; doi:10.1371/journal.pone.0038121)
Supplement: Table S4 — Results of studies not included in meta analysis. (DOC) [file pone.0038121.s010.doc]

**Supplementary Table S4**

**Results of studies not included in meta analysis**

| STUDY | TRIGLCYERIDE  mean change | P value (between groups) | CHOLESTEROL  mean change | P value | OTHER |
| --- | --- | --- | --- | --- | --- |
| Hadigan 2006 | -0.54mmol/l* -20% vs +0.65mmol/l +20% | P=0.01 | -0.07mmol/l vs  -0.02mmol/l | P=0.7 | HDL-cholesterol↑3mg/dl; LDL-cholesterol↑3mg/dl NS FFA↓0.38mEq/l (68%) p<0.001; basal rate of lipolysis ↓50% p<0.001; ↑insulin sensitivity p=0.04 |
| Chow 2010 | -0.62mmol/l* vs  -0.32mmol/l* | NR | HDL-cholesterol +0.08mmol/l* vs  -0.03mmol/l | P = 0.04 | FMD 8.4% (95%CI 6.7 to 1.1) adjusted for baseline values, in pts with HDL-cholesterol <0.9mmol/l (p=0.01) |
| Aghdassi 2010 | -0.54mmol/l (p=0.033) vs +0.12mmol/l (p=0.555) | NR | -0.28mmol/l (p=0.088) vs +0.27mmol/l (p=0.057) | NR | Sig reduction insulin resistance + blood insulin level; HOMA-IR -0.74*(-2.49,0.24) vs 0.14*(-0.64, 0.81) p=0.027 |
|  | With LD -0.78±0.30 mmol/l vs without LD 0.14±0.19 mmol/l | P=0.017 |  |  |  |
| Gerber 2008 | -3.20mmol/l* -46% (p<0.001) fish oil vs  -4.14mmol/l* -58% (p<0.001) fenofibrate | P=0.039 for % change | -0.39mmol/l -7% (p=0.028) fish oil vs  -0.56mmol/l -10% (<0.001) fenofibrate | p<0.05? | LDL-cholesterol+30mg/dl(37%) p<0.001 fish oil; Study powered for Combo Rx ↓triglyceride 65%, 23% of pts achieved <200mg/dl |
| Peabody 2002 | -13% vs -6% | NR | NR | NR |  |
| Ng 2011 | +1.32mmol/l low fat (p=0.07) vs -0.01mmol/l Mediterranean (p=0.49) | P=0.28 | +0.09mmol/l (p=0.32) +2% low fat vs  +0.52mmol/l (p=0.01) +11% Med | P=0.12 | Tricep skinfold -4.5mm (p=0.03) due to ddI/d4T use in LF gp; fat intake -33g (p=0.01) in Med gp |
| Thanasilp 2010 | NR |  | NR |  | LDL-cholesterol and HDL-cholesterol significant difference between groups |

* median values SD data estimated from information

§ unpublished data NR not reported
